# Neuroprotective Activity of Some Marine Fungal Metabolites in the 6-Hydroxydopamin- and Paraquat-Induced Parkinson’s Disease Models

**DOI:** 10.3390/md16110457

**Published:** 2018-11-21

**Authors:** Ekaterina A. Yurchenko, Ekaterina S. Menchinskaya, Evgeny A. Pislyagin, Phan Thi Hoai Trinh, Elena V. Ivanets, Olga F. Smetanina, Anton N. Yurchenko

**Affiliations:** 1 Laboratory of Bioassays and Mechanism of Action of Biologically Active Substances, G.B. Elyakov Pacific Institute of Bioorganic Chemistry Far Eastern Branch of Russian Academy of Sciences, Vladivostok 690022, Russia; ekaterinamenchinskaya@gmail.com (E.S.M.); pislyagin@hotmail.com (E.A.P.); 2 Department of Marine Biotechnology, Nhatrang Institute of Technology Research and Application, Vietnam Academy of Science and Technology, 02 Hung Vuong, Nha Trang 650000, Vietnam; phanhoaitrinh@nitra.vast.vn; 3 Graduate University of Science and Technology, Vietnam Academy of Science and Technology, 18 Hoang Quoc Viet, Cau Giay, Ha Noi 100000, Vietnam; 4 Laboratory of Chemistry of Microbial Metabolites, G.B. Elyakov Pacific Institute of Bioorganic Chemistry Far Eastern Branch of Russian Academy of Sciences, Vladivostok 690022, Russia; ev.ivanets@yandex.ru (E.V.I.); smetof@rambler.ru (O.F.S.); yurchant@ya.ru (A.N.Y.)

**Keywords:** neuroprotective activity, Parkinson’s disease, ROS, DPPH-scavenging activity, 6-OHDA, paraquat, marine fungal metabolites

## Abstract

A new melatonin analogue 6-hydroxy-*N*-acetyl-β-oxotryptamine (**1**) was isolated from the marine-derived fungus *Penicillium* sp. KMM 4672. It is the second case of melatonin-related compounds isolation from microfilamentous fungi. The neuroprotective activities of this metabolite, as well as 3-methylorsellinic acid (**2)** and 8-methoxy-3,5-dimethylisochroman-6-ol (**3**) from *Penicillium* sp. KMM 4672, candidusin A (**4**) and 4″-dehydroxycandidusin A (**5**) from *Aspergillus* sp. KMM 4676, and diketopiperazine mactanamide (**6**) from *Aspergillus flocculosus*, were investigated in the 6-hydroxydopamine (6-OHDA)- and paraquat (PQ)-induced Parkinson’s disease (PD) cell models. All of them protected Neuro2a cells against the damaging influence of 6-OHDA to varying degrees. This effect may be realized via a reactive oxygen species (ROS) scavenging pathway. The new melatonin analogue more effectively protected Neuro2A cells against the 6-OHDA-induced neuronal death, in comparison with melatonin, as well as against the PQ-induced neurotoxicity. Dehydroxylation at C-3″ and C-4″ significantly increased free radical scavenging and neuroprotective activity of candidusin-related *p*-terphenyl polyketides in both the 6-OHDA- and PQ-induced PD models.

## 1. Introduction

Currently, Parkinson’s disease is one of the most common age-related motoric neurodegenerative diseases, regardless of countries and regions. This disease is characterized clinically by resting tremor, bradykinesia, rigidity, and postural instability, that significantly worsen the life quality of patients [1]. Pathogenesis of PD includes neuronal death as a result of oxidative stress involving the increase in the intracellular level of reactive oxygen species (ROS) and reactive nitrogen species. The hyperproduction of ROS may cause several forms of cell damage, such as increasing DNA damage, lipid, and protein peroxidation which can promote mitochondrial injury. In addition, some of the studies show that ROS can cause mitochondrial dysfunction and activation of apoptosis-related death signaling, which lead to neuronal cell death. These findings show the requirement of using antioxidants as a therapeutic intervention in PD in addition to other protective agents [2]. To estimate the antioxidative properties of neuroprotective agents, both cell-free and cell assays may be used. One of the most popular cell-free tests in natural product antioxidant studies is the 2,2-diphenyl-1-picrylhydrazyl (DPPH) free radical scavenging assay [3]. A widely used cell test is ROS level determination using 2′,7′-dichlorofluorescin diacetate, that is deesterified intracellularly, and turns into the highly fluorescent 2′,7′-dichlorofluorescein upon oxidation [4].

PD etiology may be linked to several factors, including genetic susceptibility and environmental elements. Regarding environmental factors, several dopaminergic neurotoxins, including 6-hydroxydopamine (6-OHDA) and 1-methyl-4-phenyl-1,2,3,6-tetrahydropyridine (MPTP), have been identified. Moreover, some pesticides/herbicides, such as rotenone, paraquat (PQ), maneb (MB), and mancozeb (MZ), cause neurotoxicity, and induce a PD-like pathology. As a result, 6-OHDA and MPTP are common models used in PD research, and pesticide-based approaches have become secondary models of study [5,6].

It is known that marine fungi produce polyketides with antioxidant and neuroprotective properties [7,8,9]. For example, gentisyl derivatives aspergentisyls A and B, and auroglaucin-related compounds from the marine-derived *Aspergillus glaucus* exhibited a strong radical-scavenging activity in DPPH test, with an IC_50_ of 7.6–24.2 µM [7]. Terrestrol G, a dimeric derivative of gentisyl alcohol from the marine sediment-derived fungus *Penicillium terrestre*, exhibited DPPH-scavenging properties with an IC_50_ of 4.1 µM [10].

Diketopiperazines are widespread marine fungal products with different biological activities, including free radical scavenging and neuroprotection [11,12]. Gliotoxin from *Pseudallescheria* sp., neoechinulin E, and cryptoechinulin D, exhibited DPPH scavenger activity with IC_50_ values of 5.2, 46.0, and 23.6 µM, respectively [13,14]. Neoechinulin A from *Eurotium rubrum* protected PC12 cells from the cytotoxic influence of MPTP neurotoxin [15].

Some other metabolites of marine fungi also show a neuroprotective effect in the in vitro and in vivo models of Parkinson’s disease [16]. Xyloketal B, from *Xylaria* sp., scavenged free radicals in DPPH assay, and protected PC12 cells against ischemia-induced cell injury and MPTP-induced neurotoxicity [17]. About 40 synthetic derivatives of xyloketal B were investigated in various in vivo Parkinson’s models, and some of them showed significant activities [18]. Secalonic acid A from *Aspergillus ochraceus* and *Paecilomyces* sp. protected against MPTP-induced dopaminergic neuronal cell death in mouse PD model, in nigral neurons, and SH-SY5Y cells [19].

In this paper, we described the isolation and identification of new alkaloid and known polyketides from the marine fungus *Penicillium* sp. KMM 4672, known diketopiperazine alkaloid from *Aspergillus flocculosus*, polyketides reported earlier from *Aspergillus* sp. KMM 4676, as well as free radical scavenging and neuroprotective activities of these compounds in two cell models of Parkinson’s disease induced by 6-hydroxydopamine and paraquat.

## 2. Results and Discussion

### 2.1. Isolation and Identification of Compounds

**Figure 1:**
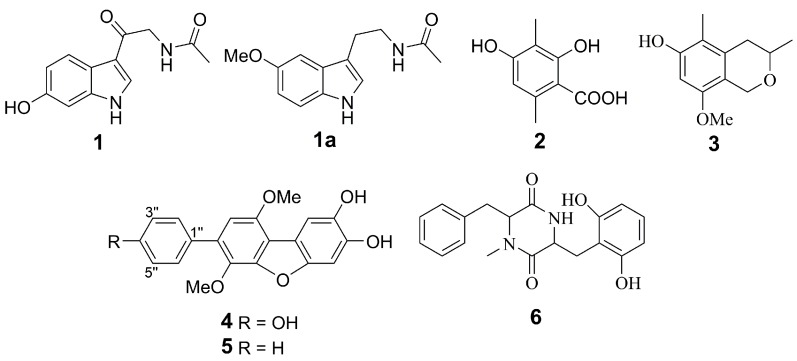
The structures of investigated compounds.

Recently, we have found new natural compounds whose structures have not been established, possibly due to their insufficient content, along with several new and known compounds from a marine algicolous fungus *Penicillium* sp. KMM 4672 [20,21]. A repeated cultivation of this fungus, in the same conditions, was carried out to obtain a sufficient amount of unidentified compounds. As a result, these compounds were identified as new melatonin derivative (**1**), known *o*-orsellinic acid (**2**) and isochromene (**3**) derivatives (Figure 1).

6-Hydroxy-*N*-acetyl-β-oxotryptamine (**1**) was isolated as a white solid. An (–)HRESIMS spectrum (Figure S8) of compound **1** contains a [M−H]^–^ pseudomolecular peak at *m*/*z* 231.0772, which indicated a molecular formula of C_12_H_12_N_2_O_3_ (calcd for C_12_H_11_N_2_O_3_,231.0775), which corresponded to six double-bond equivalents. A careful inspection of NMR data of **1** (Table 1 and Figures S1–S7) revealed the presence of one acetyl methyl, one methylene, four sp^2^-methines, four quaternary sp^2^-carbons, one keto-group, and one amide carbonyl. In addition, the ^1^H NMR spectrum contains the signal of three heteroatom protons. The coupling constant values of NH-1 (*δ*_H_ 11.55, d, *J* = 2.9 Hz), H-2 (*δ*_H_ 8.17, d, *J* = 2.9 Hz), H-4 (*δ*_H_ 7.89, d, *J* = 8.6 Hz), H-5 (*δ*_H_ 6.68, dd, *J* = 8.6, 1.7 Hz), and H-7 (*δ*_H_ 6.80, d, *J* = 1.7 Hz), together with the HMBC correlations from NH-1 to C-3a (*δ*_C_ 118.3), from H-2 to C-3a and C-7a (*δ*_C_ 137.6), from H-4 to C-3 (*δ*_C_ 114.1), C-6 (*δ*_C_ 154.0) and C-7a, from H-5 to C-3a and C-7 (*δ*_C_ 97.1), from H-7 to C-3a and C-5, and from 6-OH (*δ*_H_ 9.14) to C-5, C-6, and C-7, established the indole moiety with OH group at C-6. This suggestion was additionally proved by ROESY correlation H-1/H-7. The HMBCs from methylene H-2′ (*δ*_H_ 4.38) to C-1′ (*δ*_C_ 189.9) and C-4′ (*δ*_C_ 169.3), from H-3′ (*δ*_H_ 8.06) to C-4′, and from H-5′ (*δ*_H_ 1.90) to C-4′ showed the side chain structure. The location of the side chain at C-3 was revealed by the ROESYs of H-2′ with H-2 and H-4. Thus, the structure of **1** was elucidated to be very close to that of known *N*-acetyl-β-oxotryptamine [22]. Similar melatonin-like compounds are usually isolated from several bacterial species [23,24]. Recently, *N*-acetyl-β-oxotryptamine was reported from the medicinal basidiomycete *Inonotus vaninii* [25], and from ascomycete *Scopulariopsis* sp. [26]. To our knowledge, this study is the second case of isolation of related compounds from microfilamentous fungi.

Together with melatonin-related **1**, the well-known fungal polyketides 3-methylorsellinic acid (**2**) and 8-methoxy-3,5-dimethylisochroman-6-ol(**3**) were isolated from this fungus. Their structures were identified by comparing the NMR and MS data (Figures S9–S13) with previously reported data [27,28].

The chemical composition of extract of an ascidian-derived fungus *Aspergillus* sp. KMM 4676 was reported earlier [29,30]. *p*-Terphenyl polyketides, candidusin A (**4**) and 4″-dehydroxycandidusin A (**5**), were major metabolites of this fungus.

The 2,5-diketopiperazine alkaloid mactanamide (**6**) was isolated from the Vietnamese sediment-derived fungus *Aspergillus flocculosus*. The NMR data (Figures S14 and S15) for this compound were identical with earlier published data [31]. It is only the third case of isolation this compound.

### 2.2. Biological Activities of the Studied Compounds

#### 2.2.1. 6-Hydroxy-*N*-acetyl-β-oxotryptamine (**1**)

6-Hydroxy-*N*-acetyl-β-oxotryptamine (**1**) was not cytotoxic against neuroblastoma Neuro2a cell up to 100 µM. This compound scavenged DPPH radicals by 48% at 100 µM (Table 2).

**Figure 2:**
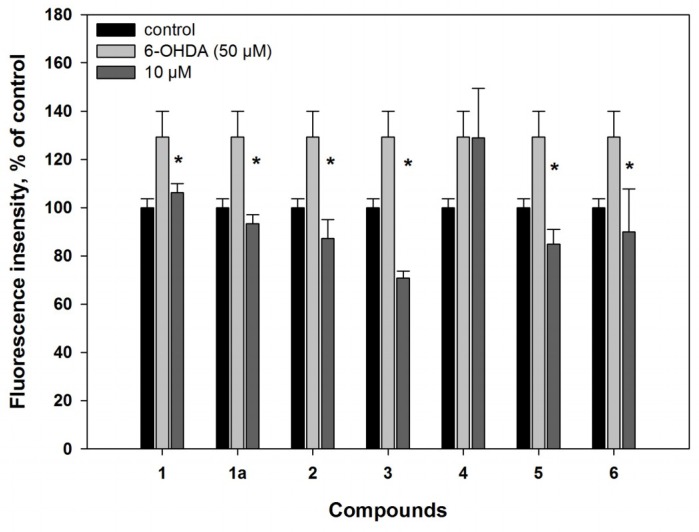
Influence of compounds **1**–**6** on reactive oxygen species (ROS) formation in Neuro2a cells treated with 6-hydroxydopamine (6-OHDA) for 30 min. * Difference between data for compounds and for 6-OHDA was statistically significant with *p* ≤ 0.05.

Melatonin-like compound **1** showed a statistically significant reduction of reactive oxygen species (ROS) level on 18% in the neuronal 6-OHDA-treated cells in the in vitro experiment (Figure 2). Melatonin (**1a**), a well-known antioxidant and neuroprotective compound, was used to compare with **1**. It decreased ROS formation in the 6-OHDA-treated neuronal cell stronger in comparison with **1**.

**Figure 3:**
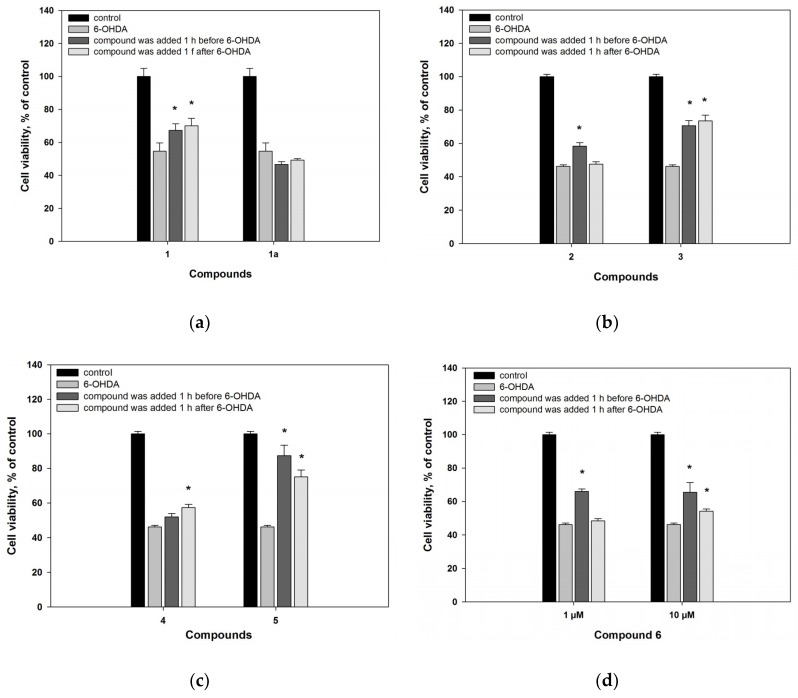
Neuroprotective effects of compounds **1**–**6** on Neuro2a cells treated with 6-OHDA (50 µM). All compounds were added to the cell suspension 1 h before treatment with 6-OHDA or 1 h after treatment with 6-OHDA. (**a**) Viability of the 6-OHDA-treated cells incubated with compounds **1** and **1a** at 10 µM; (**b**) Viability of 6-OHDA-treated cells incubated with compounds **2** and **3** at 10 µM; (**c**) Viability of 6-OHDA-treated cells incubated with compounds **4** and **5** at 10 µM; (**d**) Viability of the 6-OHDA-treated cells incubated with compound **6** (1 and 10 µM) * Difference between data for compounds and for 6-OHDA was statistically significant with *p* ≤ 0.05.

The neuroprotective effect of **1** was shown both in the Neuro2a cells treated 1 h before, as well as 1 h after, adding of 6-OHDA by 23% and 28%, respectively, at a concentration of only 10 μM. Melatonin (**1a**) did not increase the viability of cells treated with neurotoxin in this experiment (Figure 3a). Our experiments showed that 6-hydroxy-*N*-acetyl-β-oxotryptamine (**1**) more effectively protected Neuro2a cells against 6-OHDA-induced neuronal death, in comparison with melatonin (**1a**).

**Figure 4:**
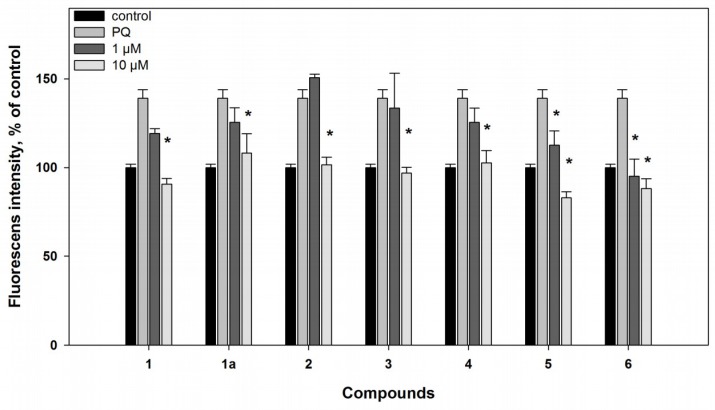
Effects of compounds **1**–**6** on ROS formation in Neuro2a cells treated with paraquat (PQ) (500 µM) for 1 h. All compounds were added to the cell suspension 1 h before treatment with PQ. * Difference between data for compounds and for PQ was statistically significant with *p* ≤ 0.05.

**Figure 5:**
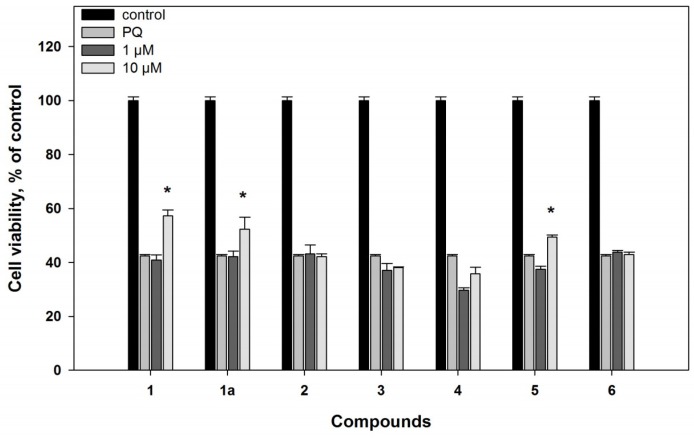
Neuroprotective effects of compounds **1**–**6** on Neuro2a cells treated with PQ (500 µM). All compounds were added to the cell suspension 1 h before treatment with PQ. * Difference between data for compounds and for PQ was statistically significant with *p* ≤ 0.05.

In the PQ-induced PD model, compounds **1** and **1a** (at concentration of 10 µM) were more effective in comparison with their influence in the 6-OHDA-induced model, and decreased ROS formation in the PQ-treated cells by 35% and 22%, respectively (Figure 4). As a result, increase of the PQ-treated cell viability, by 40% and 24%, was observed (Figure 5).

#### 2.2.2. 3-*O*-Methylorsellinic acid (**2**) and 8-methoxy-3,5-dimethylisochroman-6-ol (**3**)

3-*O*-Methylorsellinic acid (**2**) and 8-methoxy-3,5-dimethylisochroman-6-ol (**3**) were not cytotoxic against neuroblastoma Neuro2a cells up to 100 µM.

3-*O*-Methylorsellinic acid (**2**) at 100 µM scavenged 10% DPPH radicals in our experiments (Table 2). For orsellinic acid and its derivatives, 2,20-azinobis(3-ethylbenzothiozoline-6-sulfonate cation (ABTS·+) scavenger activities were recently reported [32]. 8-Methoxy-3,5-dimethylisochroman-6-ol (**3**) in DPPH assay was not very effective; also, at 100 µM concentration, it reduced the free radical value by 35%, as we reported earlier [33]. Nevertheless, in the 6-OHDA-treated Neuro2a cells, compounds **2** and **3** at a concentration of 10 µM significantly decreased ROS formation by 30% and 45% respectively (Figure 2).

3-*O*-Methylorsellinic acid (**2**) statistically significantly increased 6-OHDA-treated cell viability by 26%, when the compound was added to cells 1 h before adding 6-OHDA. When compound **2** was added to cells 1 h after adding 6-OHDA, it had no neuroprotective effect.

8-Methoxy-3,5-dimethylisochroman-6-ol (**3**) statistically significantly increased the neuroblastoma cell viability in the 6-OHDA-induced PD model by about 55%, regardless of when it was added to cells (Figure 3b). Compounds **2** and **3** were effective at a concentration of only 10 μM.

On the other hand, compounds **2** and **3** did not exhibit any protective effect on based on the viability of cells treated with PQ, despite the fact that they reduced ROS formation in these cells (Figure 4 and Figure 5).

#### 2.2.3. Candidusin A (**4**) and 4″-dehydroxycandidusin A (**5**)

Candidusin A (**4**) scavenged 32% of radicals in DPPH assay at a concentration 100 µM. 4″-Dehydroxycandidusine A (**5**) was more effective and scavenged 49% of radicals (Table 2). Candidusin B (C-3″ hydroxylated analogue of candidusin A) showed 59% DPPH radical scavenging at a concentration of 100 µg/mL [34], i.e., at a molar concentration of the compound that was more than 2.7 times higher.

Earlier, candidusin A (**4**) showed cytotoxic activities against HL-60 (IC_50_ 77.56 µM), A-549 (IC_50_ 19.34 µM), and P-388 (IC_50_ 46.83µM) tumor cells [35], while 4-dehydroxycandidusin A (**5**) exhibited cytotoxic activities against KB (IC_50_ 22.66 µM) and A 549 (IC_50_ 34.01 µM) tumor cells [36]. In our experiments, candidusin A (**4**) and 4″-dehydroxycandidusin A (**5**) had low cytotoxicity against neuroblastoma Neuro2a cells with an IC_50_ of 75.7 and 78.9 µM, respectively (Table 2). These compounds were used at a non-toxic concentration of 10 µM for the treatment of cells in PD models.

Candidusin A (**4**) did not have any effect on ROS formation in the 6-OHDA-treated cells (Figure 2). 4″-Dehydroxycandidusin A (**5**) decreased the ROS level in 6-OHDA-treated cells by 34%, being more effective as a radical scavenger. Candidusin A (**4**) had no effect on the viability of cells when it was added 1 h before treatment with 6-OHDA, and increased 6-OHDA-treated cell viability by 24% at a concentration of only 10 μM, when it was added 1 h after 6-OHDA (Figure 3c). 4″-Dehydroxycandidusin A (**5**) increased cell viability by more than 80% (when the compound was added 1 h before 6-OHDA) and 62% (when it was added 1 h after 6-OHDA). These effects were observed at a concentration of 10 μM only. When the concentration of compound **5** was reduced tenfold, its neuroprotective effect was not preserved.

In the PQ-induced model, compound **4** decreased ROS formation by 27% at a concentration of 10 µM. Compound **5** was more effective and statistically significantly decreased ROS formation by 19% and 40%, at concentrations of 1 and 10 µM, respectively (Figure 4). Nevertheless, compound **4** did not have any effect on the viability of the PQ-treated cells, but its 4″-dehydroxylated derivative (**5**) statistically significantly increased the viability of these cells by 17% at concentration of 10 µM only (Figure 5).

Thus, the presence of hydroxy groups at C-3″ and C-4″ in candidusins decreases the radical scavenging activity of these compounds in the cell-free assay. Moreover, the hydroxylation at C-4″ results in the significant decreasing their neuroprotective effect.

#### 2.2.4. Mactanamide (**6**)

Mactanamide (**6**) was not cytotoxic against neuroblastoma Neuro2a cells up to 100 µM.

Compound **6** scavenged 15% DPPH radicals in cell-free assays at a concentration of 100 µM (Table 2). In cell experiments, this compound (**6**) demonstrated a significant antiradical effect: at 10 µM, it inhibited ROS formation in the 6-OHDA-treated neuronal cells by 30% (Figure 2).

Mactanamide (**6**) increased the 6-OHDA-treated cell viability by 42% at 10 µM in the 6-OHDA-induced PD model when the compound was added 1 h before neurotoxin. When its concentration was reduced tenfold, its neuroprotective effect was preserved (Figure 3d).

In the PQ-treated cells, mactanamide (**6**) decreased ROS formation by 32% and 37%, at concentrations of 1 and 10 µM, respectively (Figure 4). However, compound **6** did not show any neuroprotective activity on the viability of the PQ-treated cells (Figure 5).

Earlier, mactanamide showed fungistatic activity against *Candida albicans*, and an influence on osteoclast differentiation without any cytotoxicity [31,37]. Antioxidant and neuroprotective properties of mactanamide were demonstrated, for the first time, in this investigation.

Thus, compounds **1**, **2**, **3**, and **6** were non-cytotoxic for Neuro2a cells up to a concentration of 100 µM. Compounds **4** and **5** demonstrated low cytotoxicity with an IC_50_ of 75.7 and 78.9 µM, respectively. This allowed investigating the neuroprotective activity of all compounds in non-toxic concentrations of 1 and 10 µM. Neuroprotective effects of the compounds were studied in two PD in vitro models using 6-OHDA and PQ as inducers of neuronal cell damage.

Melatonin-like compound **1** demonstrated an effect in increasing cell viability in both models, but the effect on PQ-treated cells was more pronounced. In both cases, neuroprotective effects were accompanied with a decrease of ROS formation in the 6-OHDA- and PQ-treated cells. Melatonin (**1a**) decreased ROS formation in both PD models, but it increased cell viability in the PQ-induced model only.

Polyketides **2** and **3** demonstrated ROS-decreasing effects in both PD models. Nevertheless, these compounds increased cell viability in the 6-OHDA-induced model only.

Candidusin A (**4**) and 4″-dehydrocandidusin A (**5**) have minimal differences between their chemical structures but this has a significant effect on their neuroprotective activity. In the 6-OHDA and PQ models, compound **5** produced a significant increase of cell viability, whereas compound **4** did not demonstrate any effect in the PQ model, and low increased cell viability on the 6-OHDA-treated cells. A similar influence of both compounds on ROS formation in the 6-OHDA- and PQ-treated cells was observed. Compound **4** had no significant effect on ROS formation in the 6-OHDA-treated cells, and decreased ROS formation in the PQ-treated cells, at a concentration of only 10 µM. By contrast, compound **5** was very effective in the 6-OHDA-induced PD model, and decreased ROS formation in the PQ-treated cells at concentrations 1 and 10 µM.

Mactanamide (**6**) demonstrated a significant decrease of ROS formation in both the 6-OHDA- and PQ-induced PD models. However, this 2,5-diketopiperasine alkaloid increased viability of the 6-OHDA-treated cells only, and did not have any statistically significant effects on viability of the PQ-treated cells.

It should be noted that DPPH radical scavenging activity was shown for all compounds in varying degrees, and decreasing of ROS formation in 6-OHDA- and PQ-treated cells could be the result of radical scavenging by these compounds. However, differences between the effects of these compounds, on ROS formation and cell viability in different PD models, were observed.

In our investigation, two PD-like cell models, induced by neurotoxin 6-OHDA and pesticide paraquat, were used. Neurotoxins and pesticides share a common mechanism to induce damage to dopaminergic neurons that is correlated with an increased oxidative status caused by high levels of ROS, anions, and free radicals [6]. However, the effect of each of the inducers, on neurons, has the same differences.

6-OHDA has a specific neurotoxic effect on neurons containing dopamine, serotonin, and norepinephrine receptors. The structure of 6-OHDA is similar to dopamine and norepinephrine, and, therefore, this neurotoxin uses the same catecholaminergic transport system (the dopamine and norepinephrine transporters), and causes specific degeneration of dopaminergic and noradrenergic neurons [6]. Inside neurons, 6-OHDA is rapidly autooxidized to hydrogen peroxide and paraquinone, which are both highly toxic to mitochondria, by specifically affecting complex I. This process results in an increase of ROS generation and cell death [38]. Moreover, it was reported that 6-OHDA induces oxidative stress both during its autoxidation to *p*-quinone and, also, during one-electron reduction of *p*-quinone to *p*-semiquinone, catalyzed by flavoenzymes that transfer one electron [39]. In addition to these effects, 6-OHDA-induced cell death is dependent of such intracellular processes as neuroinflammation, mitochondria dysfunction, endoplasmic reticulum stress, and autophagy [40].

Paraquat causes oxidative stress in neuronal cells by another pathway. Divalent paraquat ion (PQ2+) is reduced to monovalent paraquat ion (PQ+) by NADPH-oxidase of mitochondrial complex I. Subsequently, PQ+ accumulates in dopaminergic neurons and reestablishes a new redox reaction intracellularly, leading to the generation of intracellular free radicals, such as superoxide and dopamine-reactive substances. This will eventually lead to dopaminergic neuron cell death [41]. Moreover, PQ toxicity correlates with DNA fragmentation, caspase-3 cascade modulation, and dysregulation of autophagy [5,42].

Metabolic investigations of the molecular mechanisms associated with 6-OHDA and PQ toxicity were carried out by NMR spectroscopy and mass spectrometry. It was shown that PQ selectively upregulated the pentose phosphate pathway (PPP) to increase NADPH reducing equivalents, and stimulate paraquat redox cycling, oxidative stress, and cell death. PQ also stimulated an increasing in glucose uptake, the translocation of glucose transporters to the plasma membrane, and adenosine monophosphate-activated protein kinase activation. In the contract, 6-OHDA did not demonstrate an influence on PPP. In addition, while paraquat induced a reduction in glucose-dependent glutamate-derived glutathione synthesis, 6-OHDA treatment increased this process [43,44].

In this study, we observed time differences between 6-OHDA and PQ effects on ROS formation in Neuro2a cells (Figure S16). 6-OHDA caused an increase of ROS level of 30% for 30 min after addition to the cell suspension. The effect of PQ on ROS formation was insignificant after 30 min, and an increase of ROS levels in cells, by 39%, was observed 1 h after adding of PQ to the cell suspension.

Сompounds **2**, **3**, and **6** demonstrated neuroprotective effects in the 6-OHDA-induced PD model only. For this reason, they could protect Neuro2a cells against the damaging influence of products of 6-OHDA autooxidation, due to their antioxidant properties. Compound **5** increased the viability of 6-OHDA-treated cells by 80%, but it increased viability of PQ-treated cells by 17% only. This suggests the same mechanism of action.

On the other hand, compounds **1** and **1a** were more effective in the PQ-induced model, and increased cell viability by 40% and 24%, respectively, whereas in the 6-OHDA-induced model, compound **1** increased cell viability by 23% only, and melatonin (**1a**) was ineffective.

It was earlier published that melatonin and some related compounds demonstrated antioxidant activity in cell-free assays [45], and different neuroprotective effects in the in vitro experiments [46,47,48]. Pre-treating of PC12 cells with melatonin for 3 h increased viability of the cells, and prevented apoptosis in the 6-OHDA-induced PD model [49,50]. In addition, it was reported that melatonin diminished caspase-3 enzyme activity, cleavage of DNA fragmentation factor 45, and DNA fragmentation observed in the MPTP-treated neuroblastoma cells [46]. For this reason, melatonin-related compound **1** could influence on viability of the PQ-treated cells in a similar manner.

## 3. Materials and Methods

### 3.1. General Experimental Procedures

NMR spectra were recorded in DMSO-*d*_6_ and acetone-d_6_ on a Bruker DPX-500 and DRX-700 (Bruker BioSpin GmbH, Rheinstetten, Germany) spectrometers, using TMS as an internal standard. HRESIMS spectra were measured on a Maxis impact mass spectrometer (Bruker Daltonics GmbH, Rheinstetten, Germany).

Low-pressure liquid column chromatography was performed using silica gel (50/100 μm, Imid Ltd., Krasnodar, Russia). Plates (4.5 cm × 6.0 cm) precoated with silica gel (5–17 μm, Imid Ltd., Krasnodar, Russia) were used for thin-layer chromatography. Preparative HPLC was carried out on a Shimadzu LC-20 chromatograph (Shimadzu USA Manufacturing, Canby, OR, USA) with a Shimadzu RID-20A refractometer (Shimadzu Corporation, Kyoto, Japan) using a YMC ODS-AM (YMC Co., Ishikawa, Japan) (5 µm, 10 mm × 250 mm) and YMC SIL (YMC Co., Ishikawa, Japan) (5 µm, 10 mm × 250 mm) columns.

Melatonin (**1a**) was purchased from «JSC «PE «Obolenskoe» (Obolensk, Russia).

### 3.2. Fungal Strain

The strain *Penicillium* sp. KMM 4672 was isolated from brown alga *Padina* sp. (Van Phong Bay, South China Sea, Vietnam) on malt extract agar, and identified on the basis of morphological and molecular features, as described earlier [20].

The strain *Aspergillus* sp. KMM 4676 was isolated from an unidentified colonial ascidian (Shikotan Island, Pacific Ocean) on malt extract agar, and identified on the basis of morphological and molecular features as described earlier [30].

The strain *Aspergillus flocculosus* was isolated from a sediment sample (Nha Trang Bay, South China Sea, Vietnam) by inoculating on modified Sabouraud medium (peptone 10 g, glucose 20 g, agar 18 g, natural sea water 1000 mL, penicillin 1.5 g, streptomycin 1.5 g, pH 6.0–7.0). The fungus was identified according to a molecular biological protocol by DNA amplification and sequencing of the ITS region (GenBank accession number MH101466.1). BLAST search results indicated that the sequence was 100% identical (796/796 bp) with the sequence of *Aspergillus flocculosus* strain NRRL 5224 (GenBank accession number EU021616.1).

### 3.3. Cultivation of Fungus

All the fungal strains were cultured at room temperature for three weeks in 500 mL Erlenmeyer flasks, each containing rice (20.0 g), yeast extract (20.0 mg), KH_2_PO_4_ (10 mg), and natural sea water (40 mL).

### 3.4. Extraction and Isolation

The main part of the isolation procedures of compounds from *Penicillium* sp. KMM 4672 was described in a previous paper [20]. The *n*-hexane–EtOAc (95:5, 74.0 mg) fraction was purified by LH-20 column (80 cm × 2 cm) with CHCI_3_ to yield **2** (12.5 mg) and **3** (14.3 mg). The *n*-hexane–EtOAc (40:60, 54.0 mg) fraction was purified by HPLC on a YMC ODS-AM column, eluting with MeOH–H_2_O (65:35), and then by HPLC on a YMC SIL column, eluting with MeOH–CHCl_3_ (5:95) to yield **1** (1.9 mg).

The main part of isolation procedures of compounds from *Aspergillus* sp. KMM 4676 were described in a previous paper [30]. The *n*-hexane–EtOAc (80:20, 59.4 mg) fraction was purified by HPLC on a YMC ODS-AM column, eluting with MeOH–H_2_O (65:35) to yield **5** (3.45 mg). Another *n*-hexane–EtOAc (80:20, 157.7 mg) fraction was purified by HPLC on a YMC ODS-AM column, eluting with MeOH–H_2_O (65:35), and then by HPLC on a YMC SIL column, eluting with MeOH–CHCl_3_–NH_4_Ac (10:90:1) to yield **4** (6.74 mg).

The fungal mycelia of *Aspergillus flocculosus* with the medium were extracted for 24 h with 15 L of EtOAc. Evaporation of the solvent, under reduced pressure, gave a dark brown oil (5.0 g), to which 250 mL H_2_O–EtOH (4:1) was added, and the mixture was thoroughly stirred to yield a suspension. It was extracted, successively, with hexane (150 mL × 2), EtOAc (150 mL × 2), and *n*-BuOH (150 mL × 2). After evaporation of the EtOAc layer, the residual materials (3.36 g) were passed over a silica gel column (35.0 cm × 2.5 cm), which was eluted with a hexane–EtOAc gradient (1:0–0:1). The *n*-hexane–EtOAc (75:25, 41.4 mg) fraction was purified by HPLC on a YMC SIL column, eluting with MeOH–CHCl_3_-NH_4_Ac (97:3:1), and then by HPLC on a YMC ODS-AM column, eluting with MeOH–H_2_O (75:25) to yield **6** (24.1 mg).

6-Hydroxy-*N*-acetyl-β-oxotryptamine (**1**): white powder; ^1^H and ^13^C NMR data see Table 1, Figures S1–S7; HR ESIMS *m*/*z* 231.0772 [M−H]^–^ (calcd for C_12_H_11_N_2_O_3_, 231.0775, Δ + 1.4 ppm) (Figure S8).

### 3.5. Biological Activity of Compounds

#### 3.5.1. Radical Scavenger Assay

DPPH radical scavenging activity of compounds was tested as described [51].

Compounds were dissolved in MeOH, and the solutions (160 µL) were dispensed into wells of a 96-well microplate. In all, 40 µL of the DPPH (Sigma-Aldrich, Steinheim, Germany) solution in MeOH (1.5 × 10^−4^ M) was added to each well. Concentrations of compounds in mixture were 10 and 100 µM. The mixture was shaken and left to stand for 30 min, and the absorbance of the resulting solution was measured at 520 nm with a microplate reader MultiscanFC (ThermoScientific, Waltham, MA, USA). Radical scavenging activity of all compounds at 100 µM were presented as percent of MeOH data, and the concentration of DPPH radical scavenging at 50% (EC_50_) was calculated for some compounds.

#### 3.5.2. Cell Line and Culture Condition

The neuroblastoma cell line Neuro2a was purchased from ATCC. Cells were cultured in DMEM medium containing 10% fetal bovine serum (Biolot, St. Petersburg, Russia) and 1% penicillin/streptomycin (Invitrogen, Carlsbad, CA, USA). Cells were incubated at 37 °C in a humidified atmosphere containing 5% (*v*/*v*) CO_2_.

#### 3.5.3. Cell Viability Assay

Cell suspension (1 × 10^3^ cells/well) was incubated with different concentration of compounds for 24 h. After that, cell viability was determined using the MTT (3-(4,5-dimethylthiazol-2-yl)-2,5- diphenyltetrazolium bromide) method, according to the manufacturer’s instructions (Sigma-Aldrich, St. Louis, MO, USA). The results were presented as percent of control data, and concentration required for 50% inhibition of cell viability (IC_50_) was calculated.

#### 3.5.4. 6-Hydroxydopamine-Induced In Vitro Model of Parkinson’s Disease

The neuroprotective activities of compounds in 6-hydroxydopamine-induced cell model of Parkinson’s disease were examined, as described previously [52].

Neuroblastoma Neuro2a line cells (1 × 10^3^ cells/well) were treated with 50 µM of 6-hydroxydopamine (Sigma-Aldrich, St. Louis, MO, USA) for 1 h and, after that, the investigated compounds were added to the neuroblastoma cell suspension at a concentration of 1 and 10 µM. In the other case, the substances were added to the cells 1 h before the addition of the neurotoxin. Cells incubated without 6-OHDA and compounds, and with 6-OHDA only, were used as positive and negative controls, respectively. After 24 h, viability of cells was measured using the MTT method. The results were presented as a percent of positive control data.

#### 3.5.5. Paraquat-Induced In Vitro Model of Parkinson’s Disease

Neuroblastoma Neuro2a line cells (1 × 10^3^ cells/well) were treated with compounds at concentrations of 1 and 10 µM for 1 h, and then 500 µM of paraquat (Sigma-Aldrich, St. Louis, MO, USA) was added to the neuroblastoma cell suspension. Cells incubated without paraquat and compounds, and with paraquat only, were used as positive and negative controls, respectively. The viability of cells was measured after 24 h using the MTT method. The results were presented as percent of positive control data.

#### 3.5.6. Reactive Oxygen Species (ROS) Level Analysis in 6-OHDA- and PQ-Treated Cells

Cell suspensions (1 × 10^3^ cells/well) were incubated with compound solutions (10 µM) for 1 h. Then, 6-OHDA at a concentration of 50 µM was added in each well, and cells were incubated for 30 min. In other experiments, cells were incubated with PQ at a concentration of 500 µM for 30 min and 1 h. Cells incubated without 6-OHDA/PQ and compounds, and with 6-OHDA/PQ only, were used as positive and negative controls, respectively. To study ROS formation, 20 µL of 2,7-dichlorodihydrofluorescein diacetate solution (Molecular Probes, Eugene, OR, USA) was added to each well, such that the final concentration was 10 mM, and the microplate was incubated for an additional 10 min at 37 °C. The intensity of dichlorofluorescein fluorescence was measured with plate reader PHERAstar FS (BMG Labtech, Ortenberg, Germany) at λ_ex_ = 485 nm, and λ_em_ = 518 nm. The data were processed by MARS Data Analysis v. 3.01R2 (BMG Labtech, Ortenberg, Germany). The results were presented as a percentage of positive control data.

## 4. Conclusions

This study is the second case of isolation of melatonin-related compound from microfilamentous fungi. The neuroprotective activity in the 6-OHDA- and PQ-induced PD cell models of this and some other polyketides and alkaloids from marine-derived fungi were investigated. All of them protected Neuro2a cells against the damaging influence of 6-OHDA to varying degrees. We suppose that this effect is realized via a ROS scavenging pathway as one of the possibilities. The new melatonin analogue, 6-hydroxy-*N*-acetyl-β-oxotryptamine, protected Neuro2A cells more effectively against the 6-OHDA-induced neuronal death in comparison with melatonin. Moreover, 6-hydroxy-*N*-acetyl-β-oxotryptamine and melatonin protected Neuro2a cells against the damaging influence of PQ in a similar manner. It was shown that dehydroxylation at C-3″ and C-4″significantly increases free radical scavenging and neuroprotective activity of candidusin-related *p*-terphenyl polyketides in both the 6-OHDA- and PQ-induced PD models.

## Figures and Tables

**Table 1:** ^1^H and^13^С NMR data (700/176 MHz, *δ* in ppm, DMSO-*d*_6_) for 6-hydroxy-*N*-acetyl-β-oxotryptamine (**1**).

Position	*δ*_С_, mult	*δ*_H_ (*J* in Hz)
1(NH)		11.55, d (2.9)
2	131.9, CH	8.17, d (2.9)
3	114.1, C	
3a	118.3, C	
4	121.5, CH	7.89, d 8.6)
5	111.9, CH	6.68, dd (8.6, 1.7)
6	154.0, C	
7	97.1, CH	6.80, d (1.7)
7a	137.6, C	
1′	189.9, C	
2′	45.4, CH_2_	4.38, d (5.6)
3′(NH)		8.06, d (5.6)
4′	169.3, C	
5′	22.4, CH_3_	1.90, s
6-OH		9.14, s

**Table 2:** Radical scavenging and cytotoxicity activities of compounds **1**–**6**.

Compounds	DPPH Radical Scavenging		Cytotoxicity
	% at 100 µM	EC_50_, µM	IC_50_, µM
**1**	51.9 ± 1.3	-	>100
**2**	88.9 ± 2.9	-	>100
**3**	62.0 ± 4.6 [16]	-	>100
**4**	67.8 ± 1.1	-	75.7 ± 5.6
**5**	50.6 ± 1.3	101.3 ± 2.8	78.9 ± 1.9
**6**	83.5 ± 0.2	-	>100

## Supplementary Materials

The following are available online at http://www.mdpi.com/1660-3397/16/11/457/s1, Figures S1–S15. The NMR and mass spectra of compounds **1**–**3** and **6**. Figure S16. ROS formation in the 6-OHDA- and PQ-treated Neuro2a cells.
