# Supplementary material for: Neuroprotective Activity of Some Marine Fungal Metabolites in the 6-Hydroxydopamin- and Paraquat-Induced Parkinson’s Disease Models
Source: Mar Drugs. 2018 Nov 21;16(11):457. doi: 10.3390/md16110457 (PMC6265791; doi:10.3390/md16110457)

Supplementary data

Neuroprotective activity of some marine fungal metabolites in the 6-hydroxydopamin- and paraquat-induced Parkinson's disease models

Ekaterina A. Yurchenko [1,*], Ekaterina S. Menchinskaya [1], Evgeny A. Pislyagin [1], Phan Thi Hoai Trinh [2,3], Elena V. Ivanets [4], Olga F. Smetanina [4] and Anton N. Yurchenko [4]

[1] Laboratory of bioassays and mechanism of action of biologically active substances, G.B.Elyakov Pacific Institute of Bioorganic Chemistry Far Eastern Branch of Russian Academy of Sciences, Vladivostok, 690022, Russian Federation; dminae@mail.ru (E.A.Yu.); ekaterinamenchinskaya@gmail.com (E.S.M.); pislyagin@hotmail.com (E.A.P.)

[2] Department of Marine Biotechnology, Nhatrang Institute of Technology Research and Application, Vietnam Academy of Science and Technology, 02 Hung Vuong, Nha Trang, Vietnam; phanhoaitrinh@nitra.vast.vn (P.T.H.T.)

[3] Graduate University of Science and Technology, Vietnam Academy of Science and Technology, 18 Hoang Quoc Viet, Cau Giay, Ha Noi, Vietnam;

[4] Laboratory of chemistry of microbial metabolites, G.B. Elyakov Pacific Institute of Bioorganic Chemistry Far Eastern Branch of Russian Academy of Sciences, Vladivostok, 690022, Russian Federation; yurchant@ya.ru (A.N.Yu.); ev.ivanets@yandex.ru (E.V.I.); smetof@rambler.ru (O.F.S.);

* Correspondence: dminae@mail.ru; Tel.: +7-4232-318832

Content

Figure S1. ^{1}H NMR (700 MHz, DMSO-d_6) spectrum of 6-hydroxy-N-acetyl-β-oxotryptamine (**1**) 3

Figure S2. ^{13}C NMR (125 MHz, DMSO-d_6) spectrum of 6-hydroxy-N-acetyl-β-oxotryptamine (**1**) 4

Figure S3. DEPT-135 (125 MHz, DMSO-d_6) spectrum of 6-hydroxy-N-acetyl-β-oxotryptamine (**1**)....... 5

Figure S4. HSQC (700 MHz, DMSO-d_6) spectrum of 6-hydroxy-N-acetyl-β-oxotryptamine (**1**)............ 6

Figure S5. HMBC (700 MHz, DMSO-d_6) spectrum of 6-hydroxy-N-acetyl-β-oxotryptamine (**1**) 7

Figure S6. COSY (700 MHz, DMSO-d_6) spectrum of 6-hydroxy-N-acetyl-β-oxotryptamine (**1**).............. 8

Figure S7. ROESY (700 MHz, DMSO-d6) spectrum of 6-hydroxy-N-acetyl-β-oxotryptamine (**1**).................. 9

Figure S8. ESI mass spectra of 6-hydroxy-N-acetyl-β-oxotryptamine (**1**).. 10

Figure S9. ^{1}H NMR (700 MHz, acetone-d_6) spectrum of 3-methylorsellinic acid (**2**) 11

Figure S10. ^{13}C NMR (125 MHz, acetone-d_6) spectrum of 3-methylorsellinic acid (**2**)........................... 12

Figure S11. ESI mass spectra of 3-methylorsellinic acid (**2**)... 13

Figure S12. ^{1}H NMR (700 MHz, DMSO-d_6) spectrum of 8-methoxy-3,5-dimethylisochroman-6-ol (**3**)... 14

Figure S13. ^{13}C NMR (176 MHz, DMSO-d_6) spectrum of 8-methoxy-3,5-dimethylisochroman-6-ol (**3**)... 15

Figure S14. ^{1}H NMR (500 MHz, acetone-d_6) spectrum of mactanamide (**6**).. 16

Figure S15. ^{13}C NMR (125 MHz, acetone-d_6) spectrum of mactanamide (**6**)... 17

Figure S16. ROS formation in 6-OHDA- and PQ-treated Neuro2a cell... 18

Figure S1. ^{1}H NMR (700 MHz, DMSO-d_6) spectrum of 6-hydroxy-N-acetyl-β-oxotryptamine (**1**)

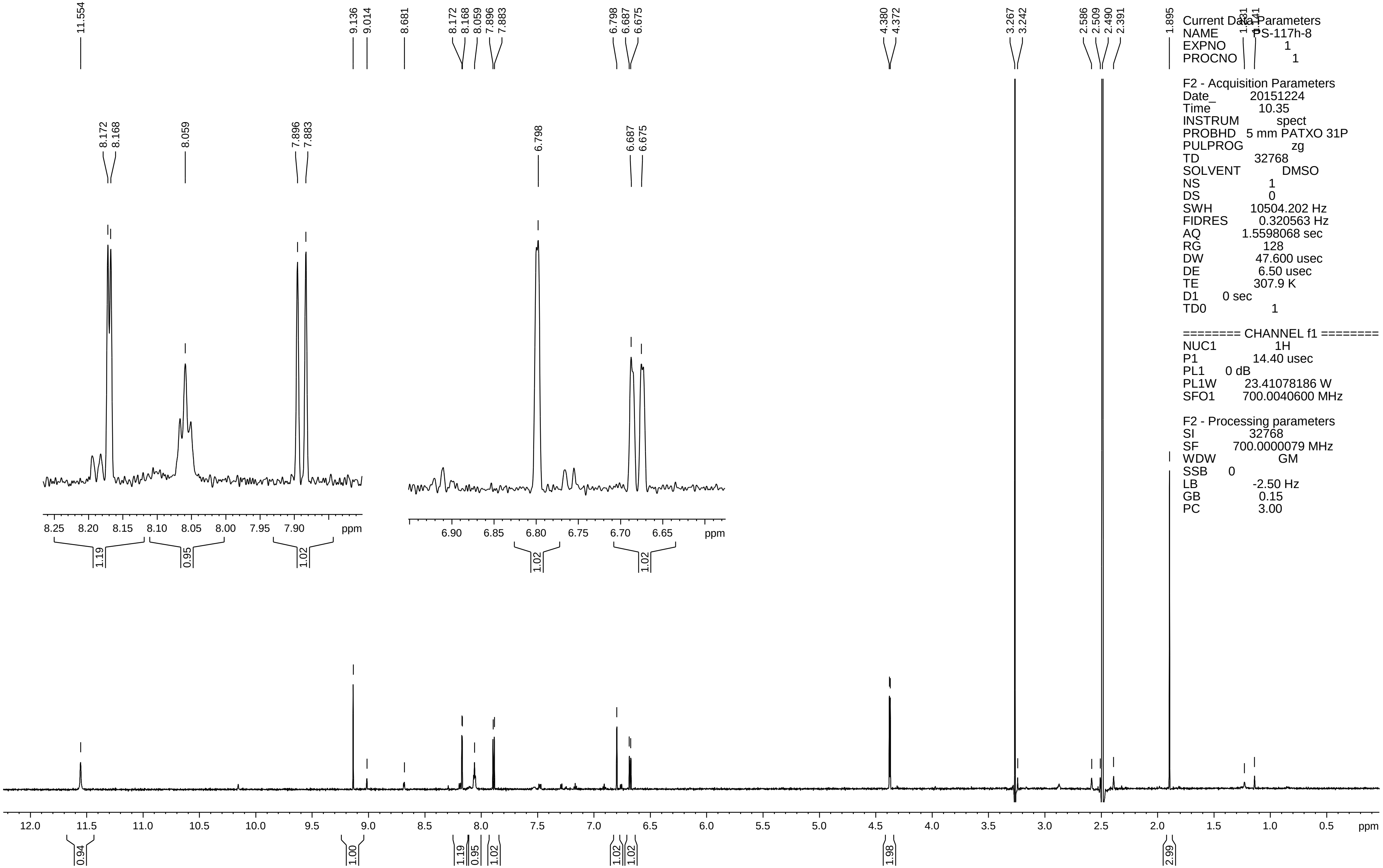

Figure S2. ^{13}C NMR (176 MHz, DMSO-d_6) spectrum of 6-hydroxy-N-acetyl-β-oxotryptamine (**1**)

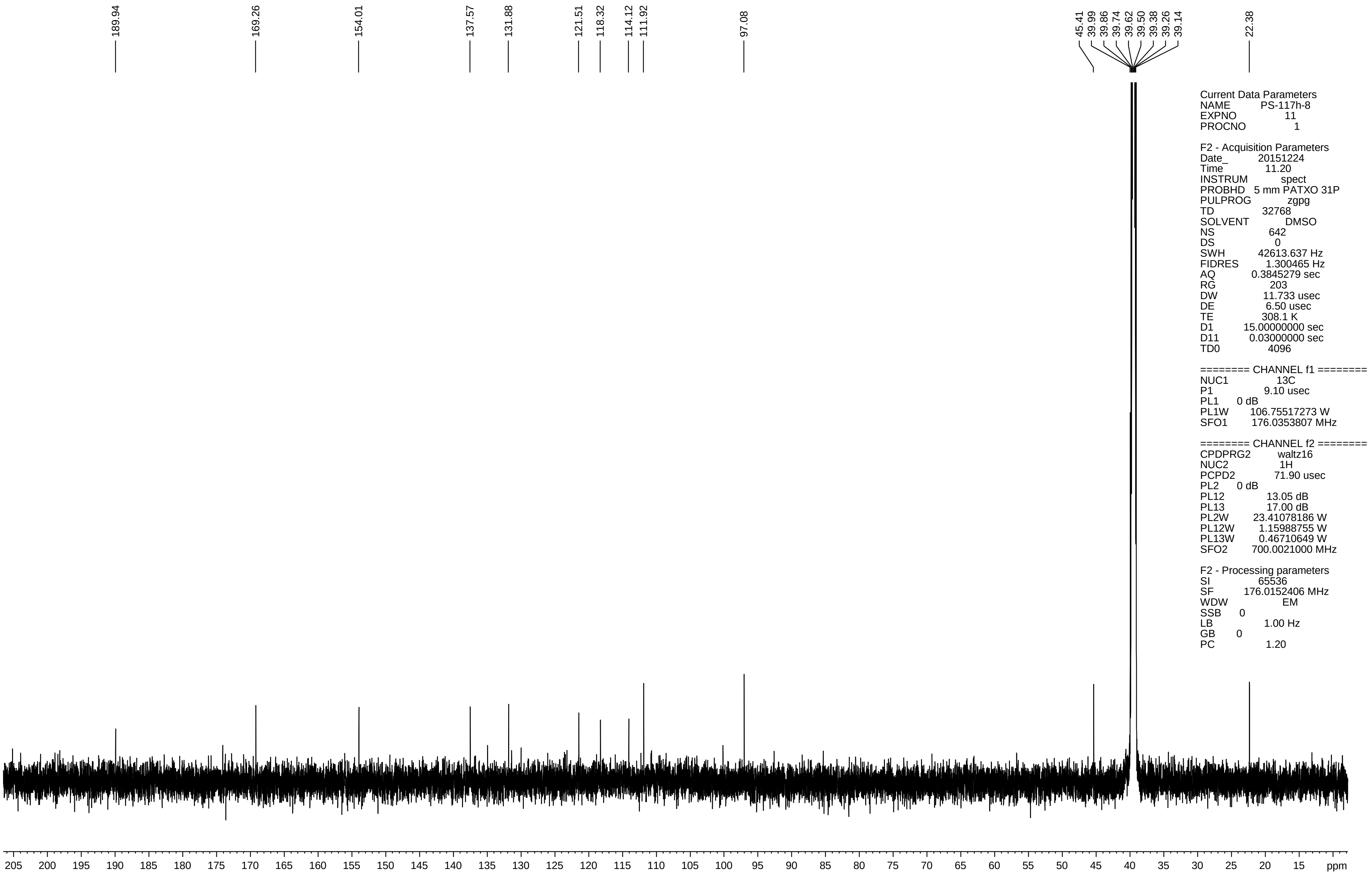

Figure S3. DEPT-135 (176 MHz, DMSO-d_6) spectrum of 6-hydroxy-N-acetyl-β-oxotryptamine (**1**)

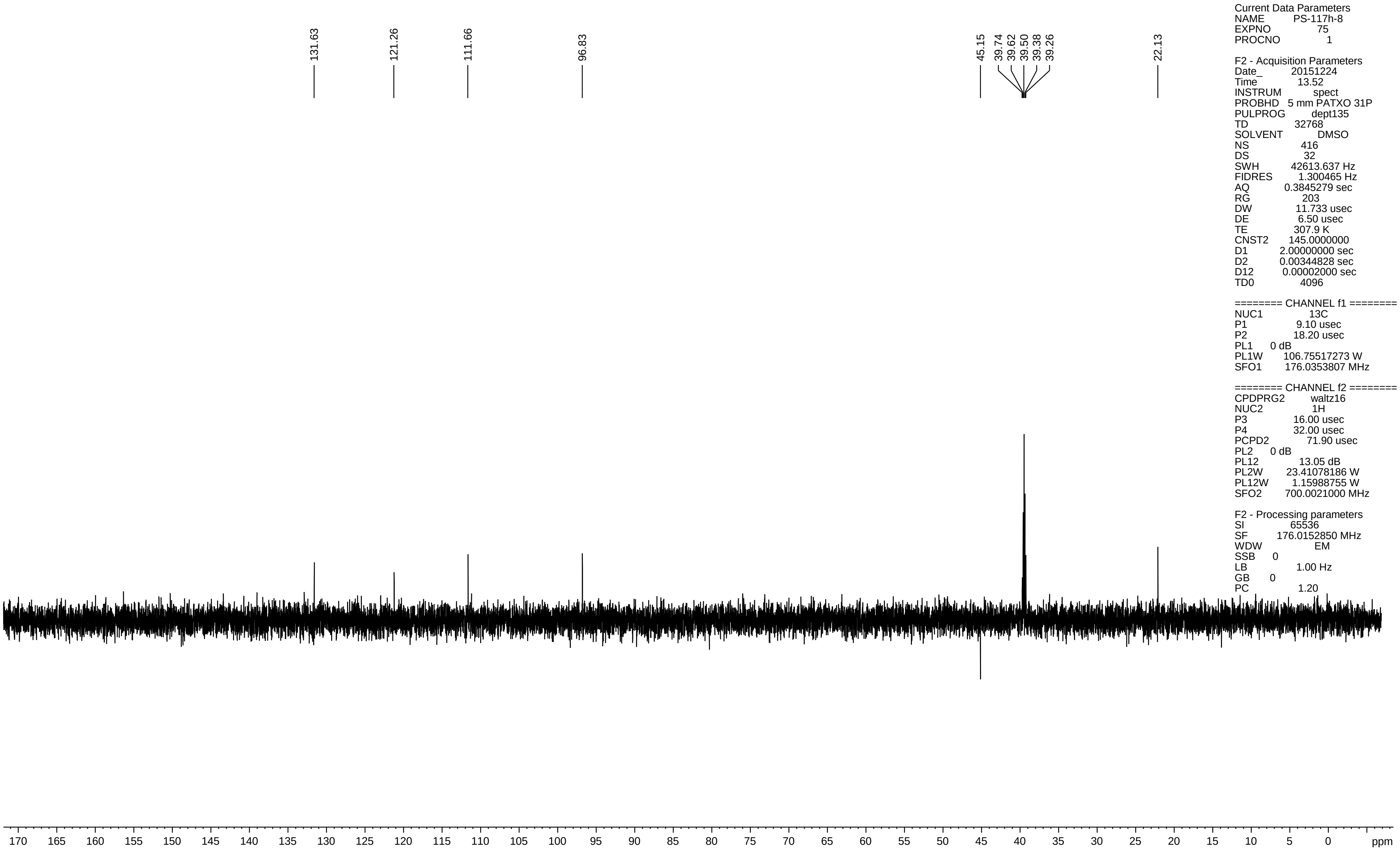

Figure S4. HSQC (700 MHz, DMSO-d$_6$) spectrum of 6-hydroxy-N-acetyl-β-oxotryptamine (**1**)

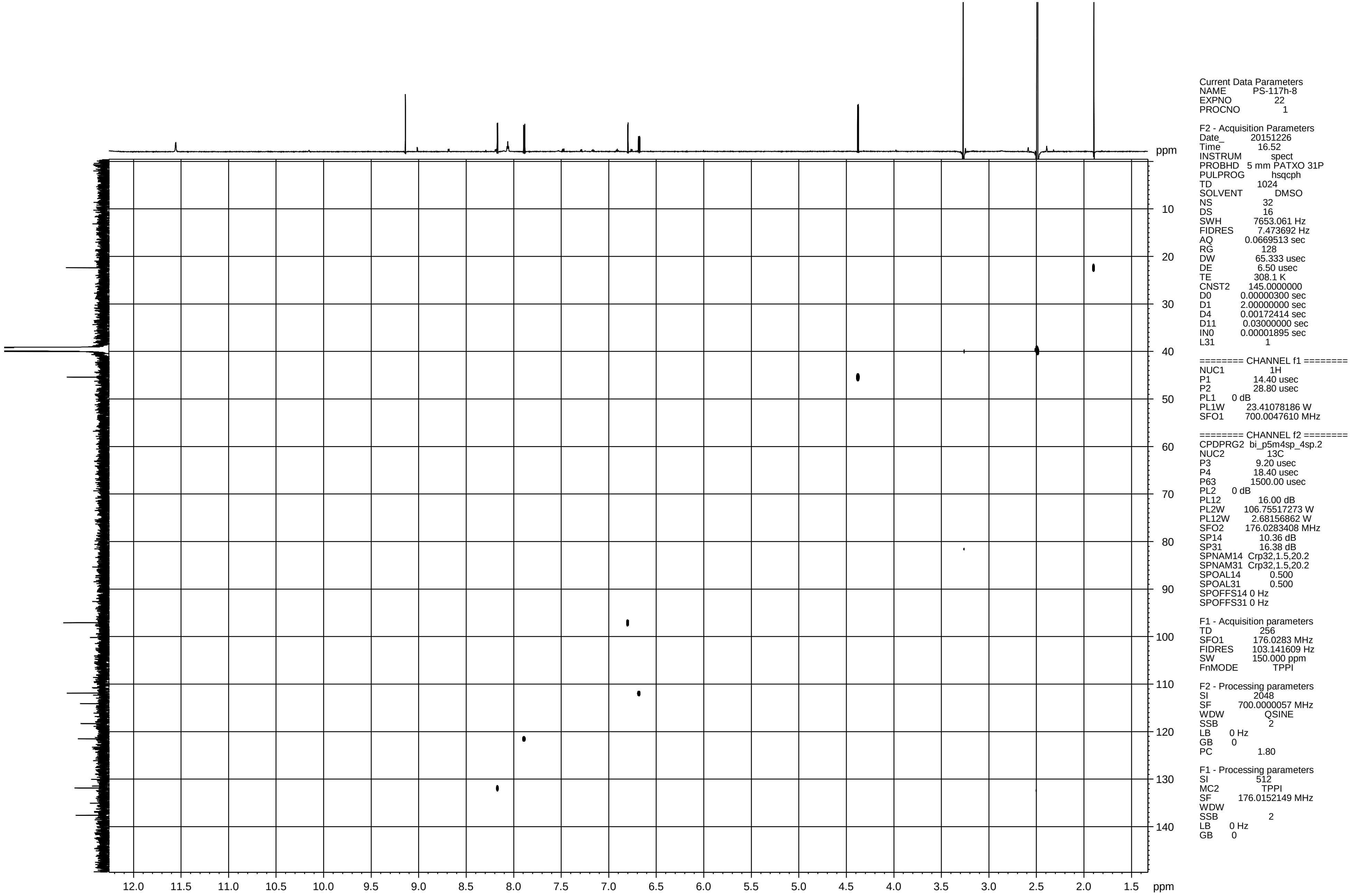

Figure S5. HMBC (700 MHz, DMSO-d_6) spectrum of 6-hydroxy-N-acetyl-β-oxotryptamine (**1**)

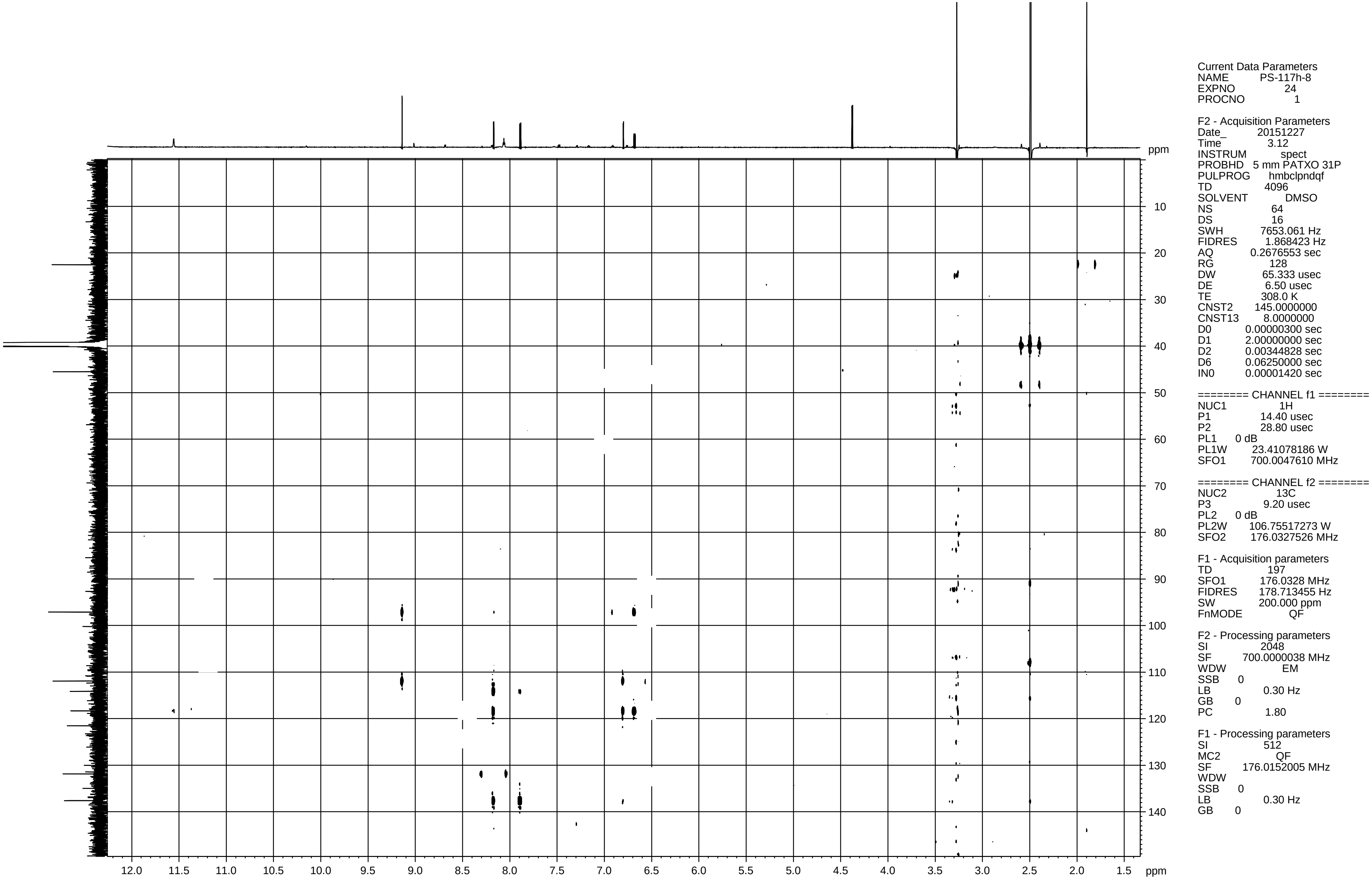

Figure S6. COSY (700 MHz, DMSO-d_6) spectrum of 6-hydroxy-N-acetyl-β-oxotryptamine (**1**)

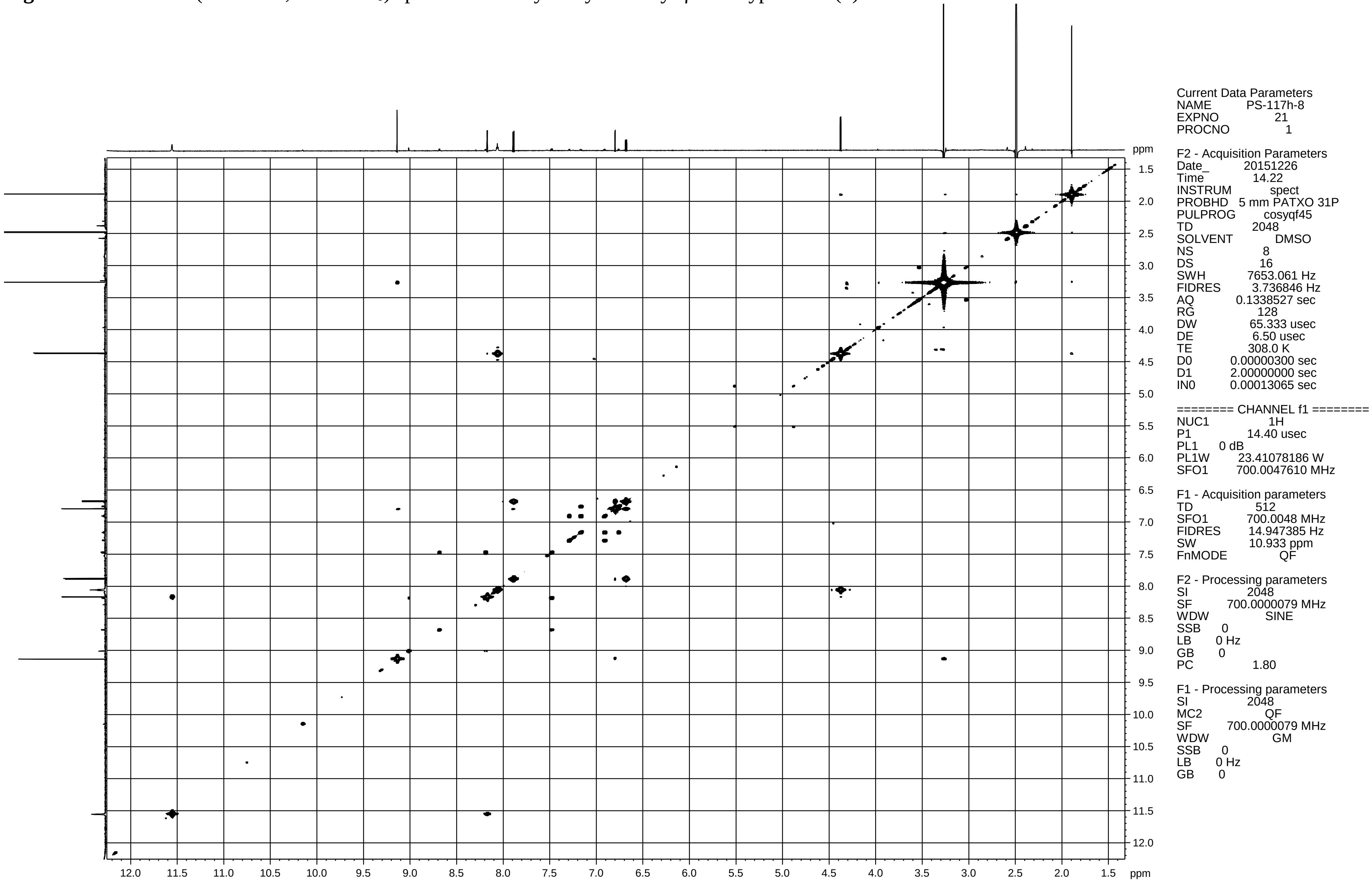

Figure S7. ROESY (700 MHz, DMSO-d_6) spectrum of 6-hydroxy-N-acetyl-β-oxotryptamine (**1**)

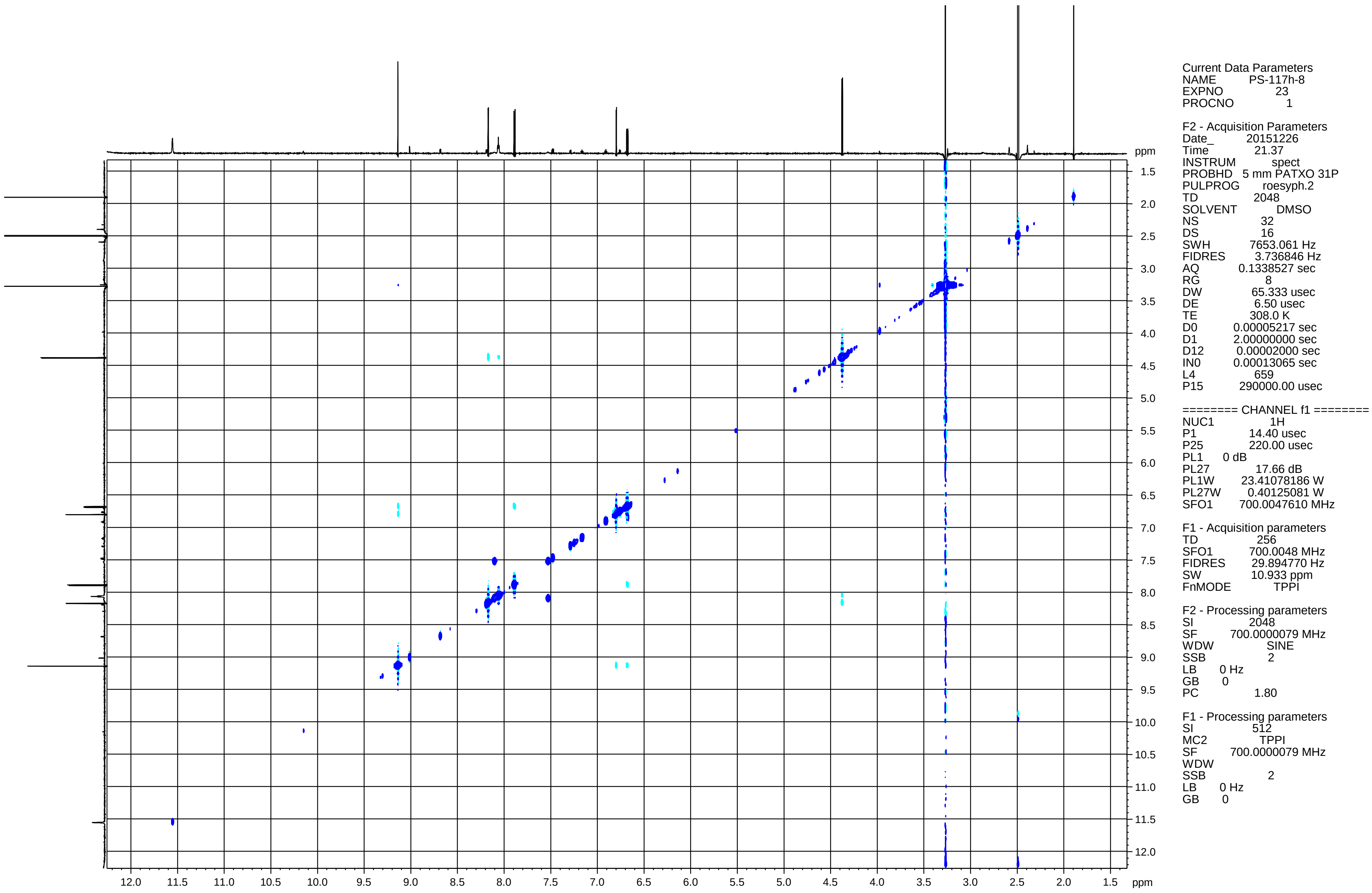

Figure S8. ESI mass spectra of 6-hydroxy-N-acetyl-β-oxotryptamine (**1**)

(–)-ESI

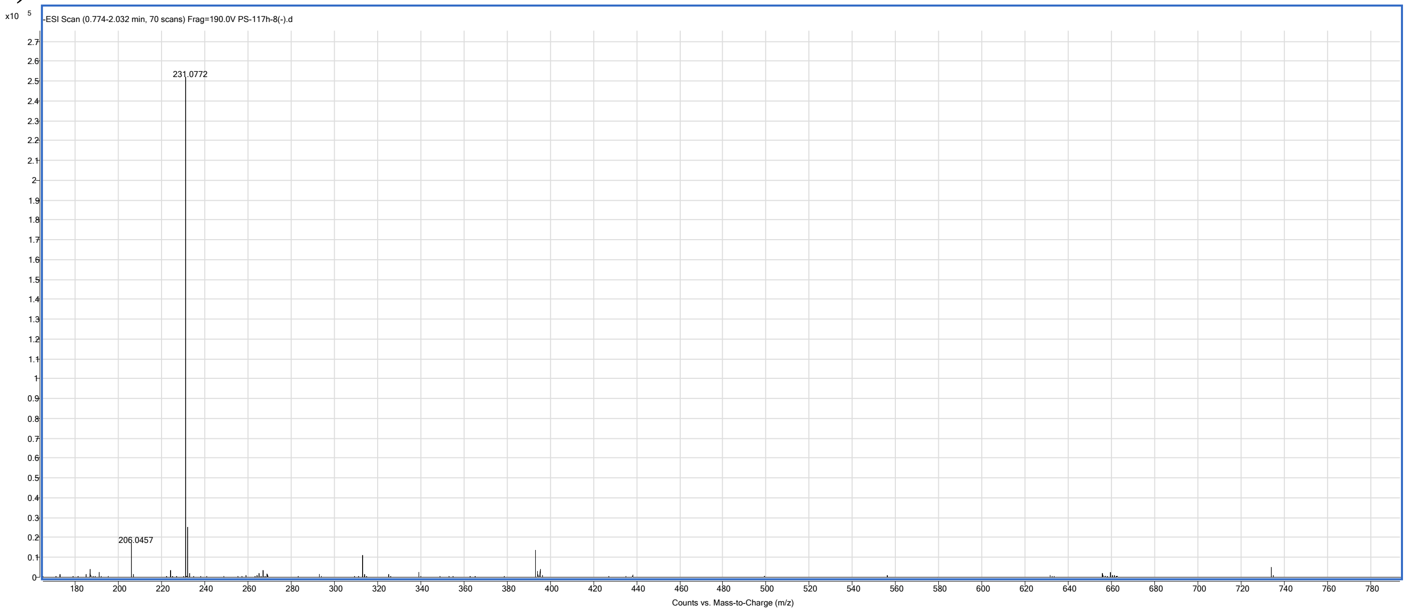

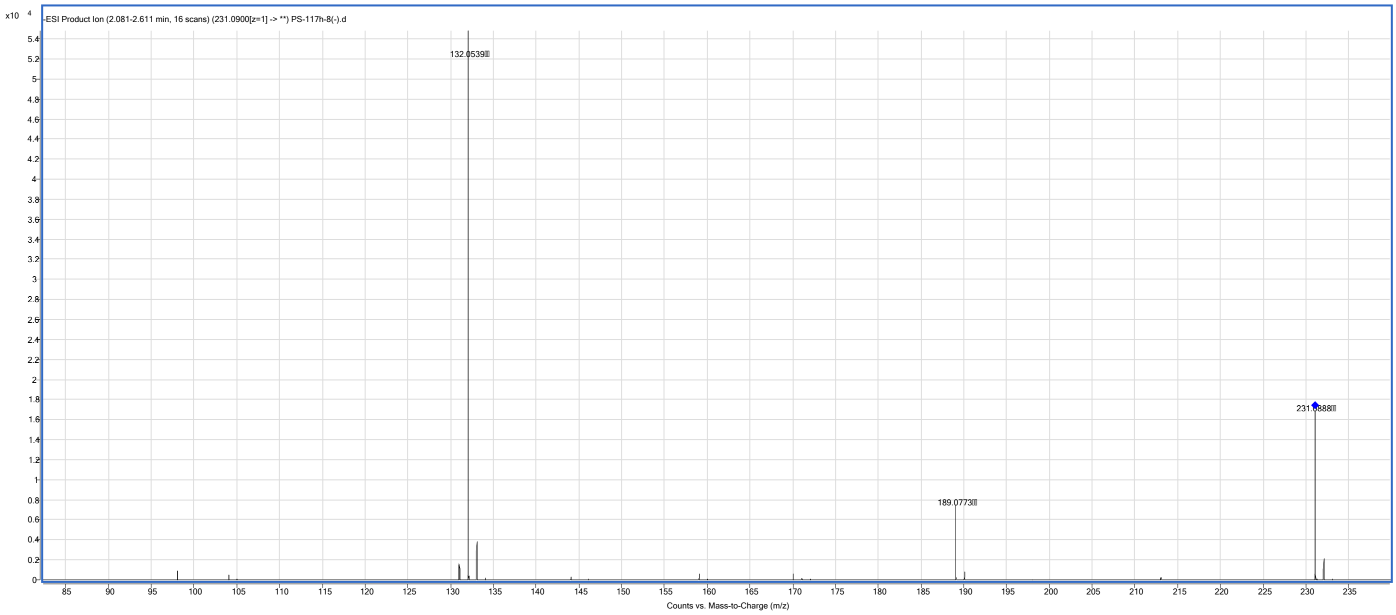

Figure S9. ^{1}H NMR (700 MHz, acetone-d_6) spectra of 3-methylorsellinic acid (**2**)

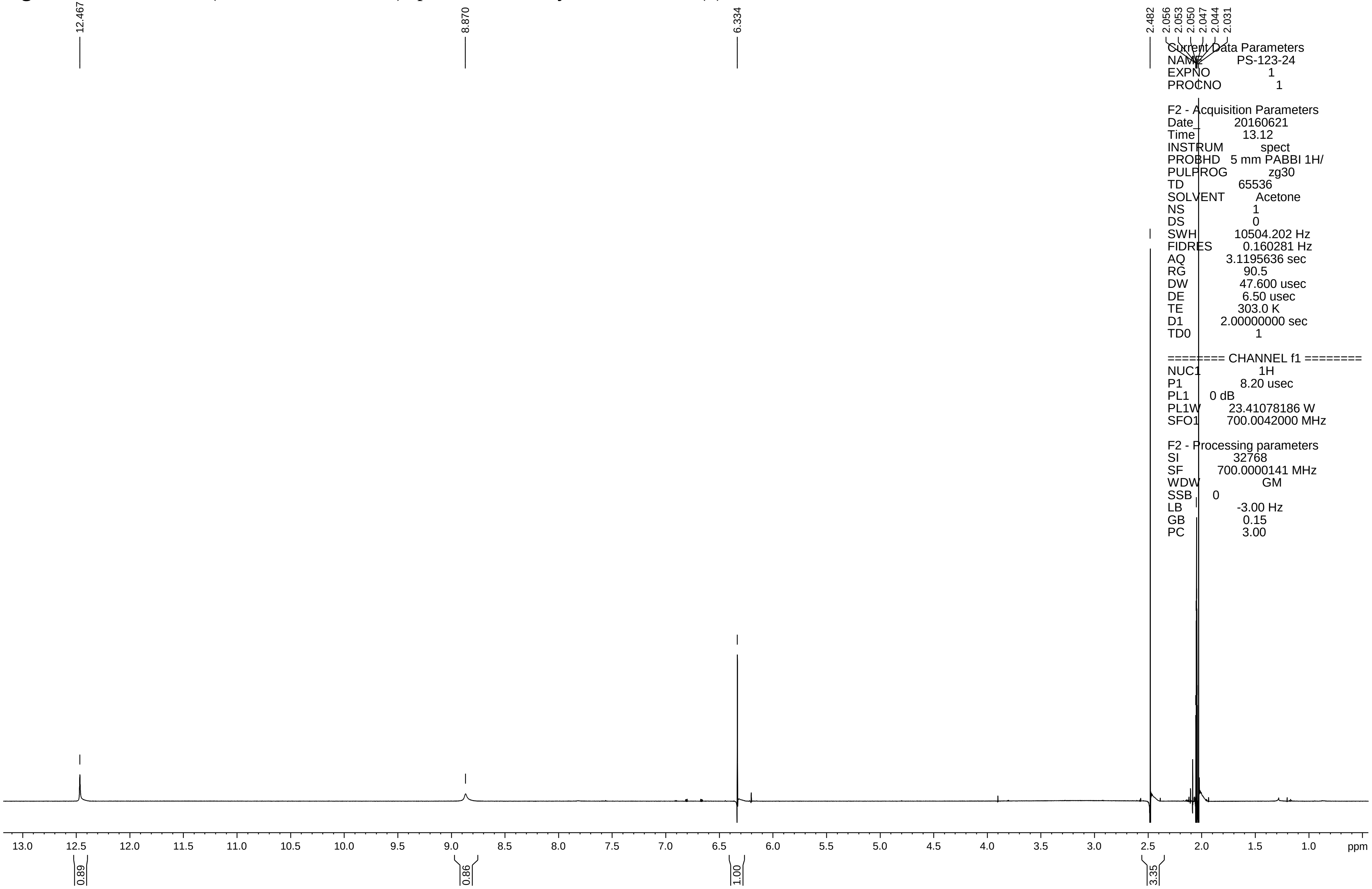

Figure S10. ^{13}C NMR (125 MHz, acetone-d_6) spectrum of 3-methylorsellinic acid (**2**)

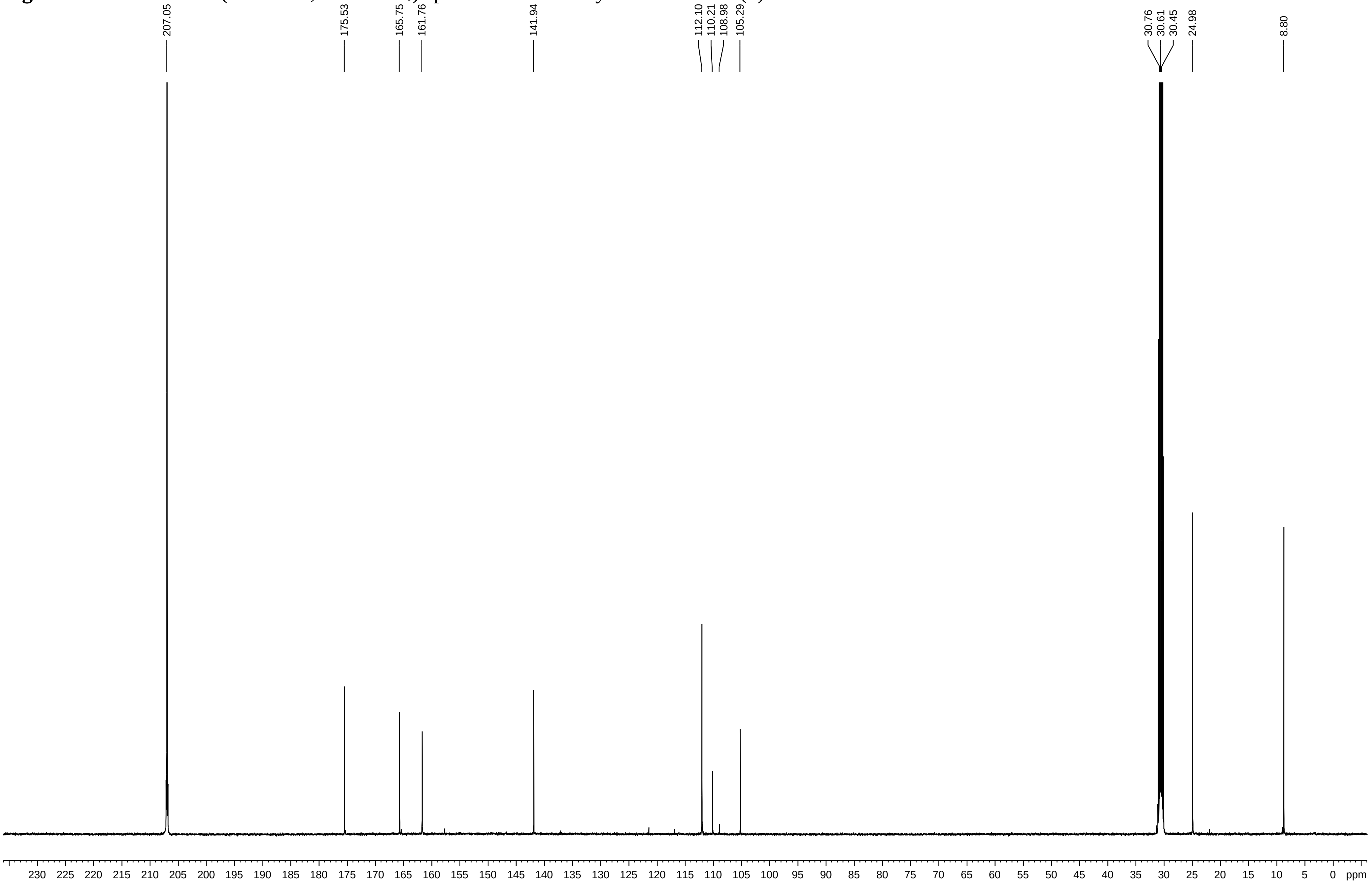

Figure S11. ESI mass spectra of 3-methylorsellinic acid (**2**)

(–)-ESI

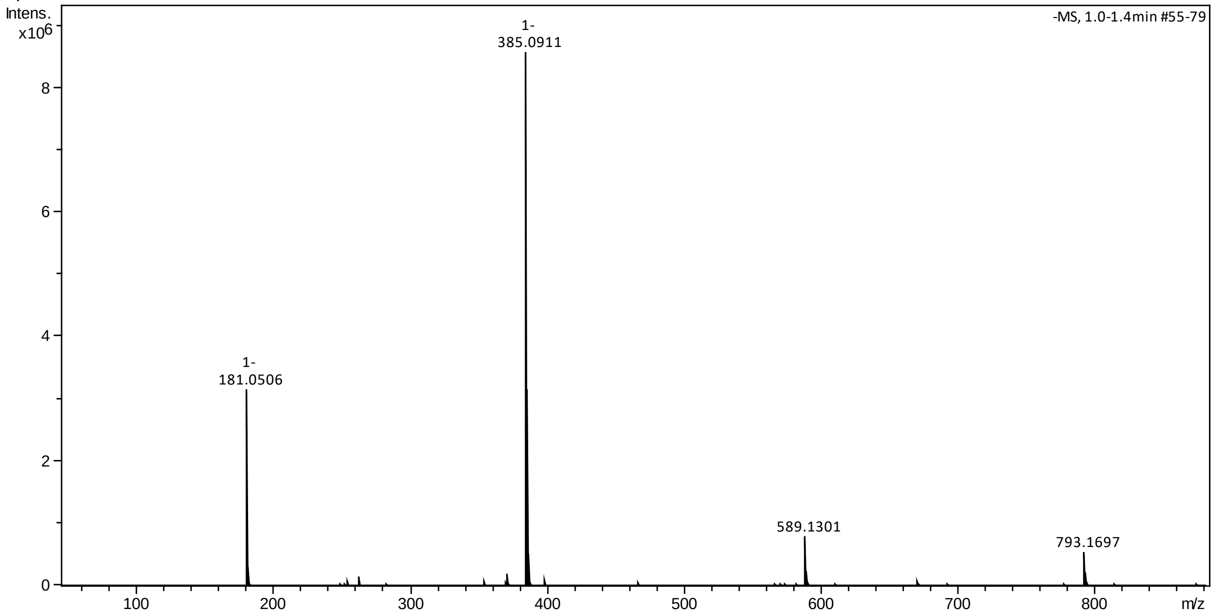

MS/MS

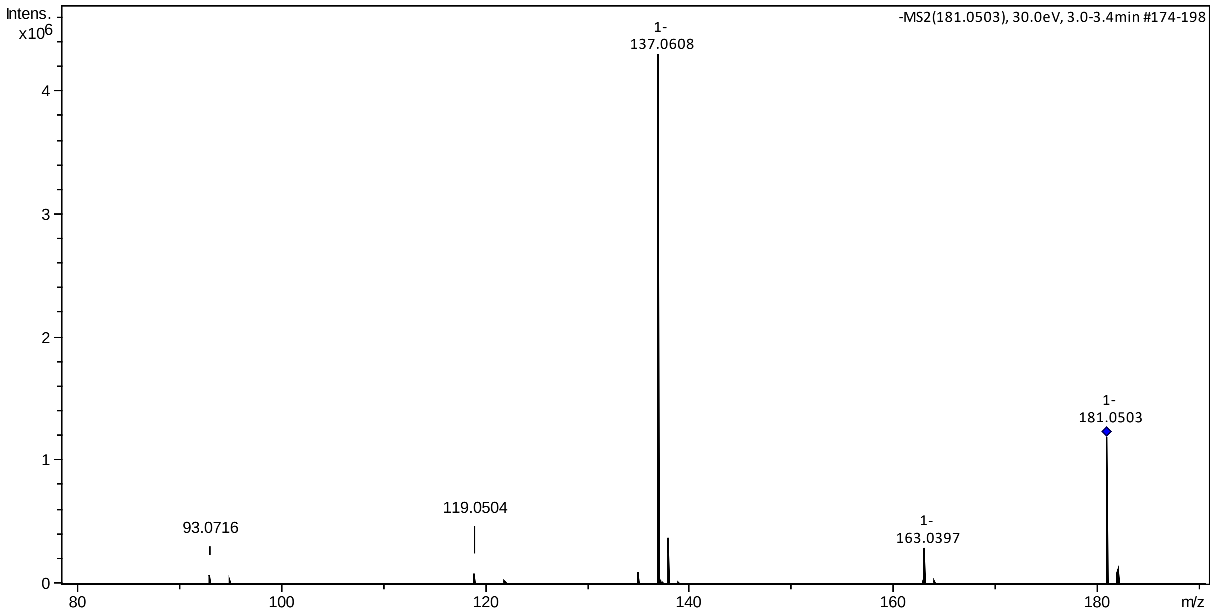

Figure S12. ^{1}H NMR (700 MHz, DMSO-d$_6$) spectra of 8-methoxy-3,5-dimethylisochroman-6-ol **(3)**

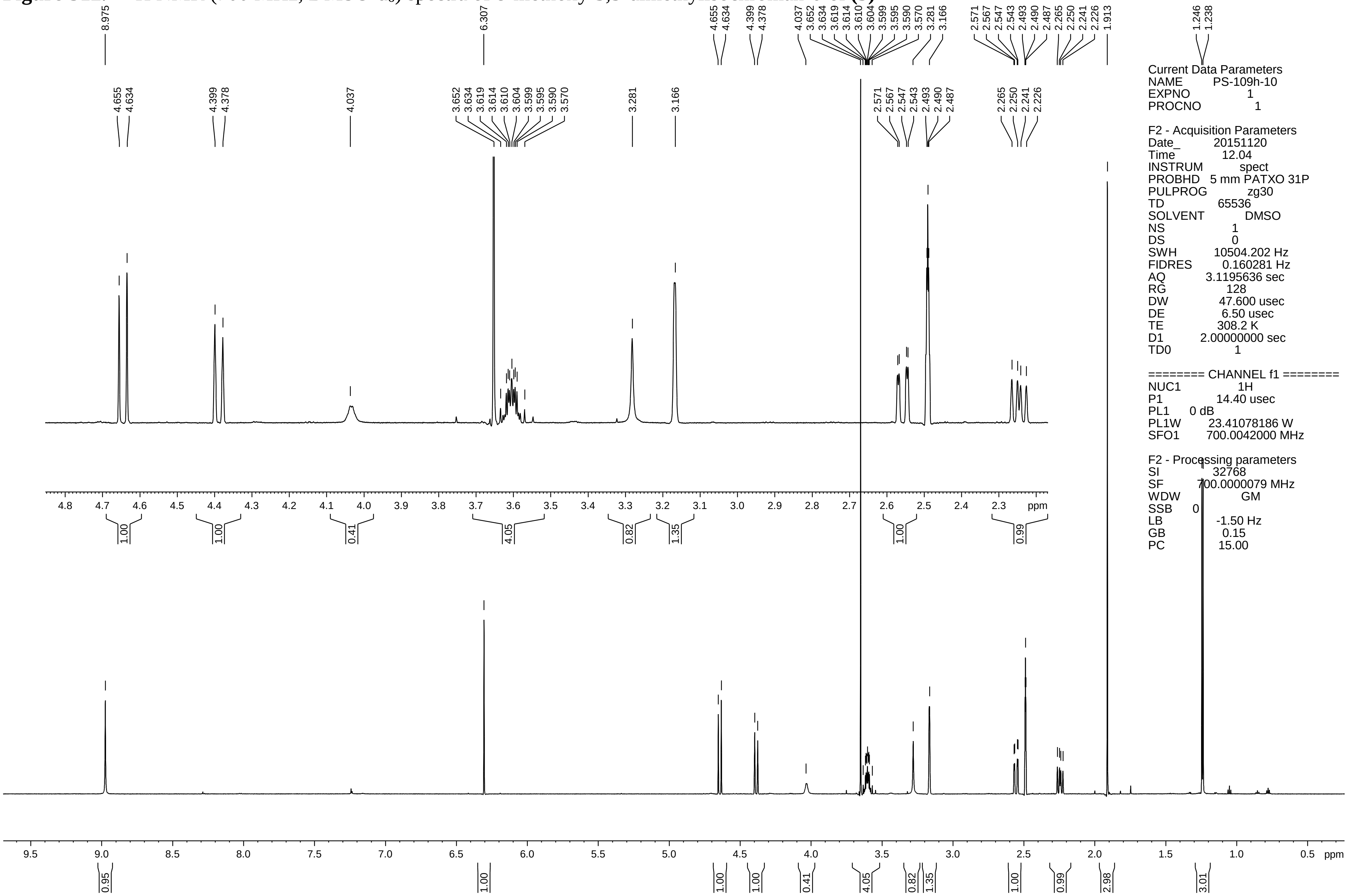

Figure S13. ^{13}C NMR (176 MHz, DMSO-d_6) spectra of 8-methoxy-3,5-dimethylisochroman-6-ol **(3)**

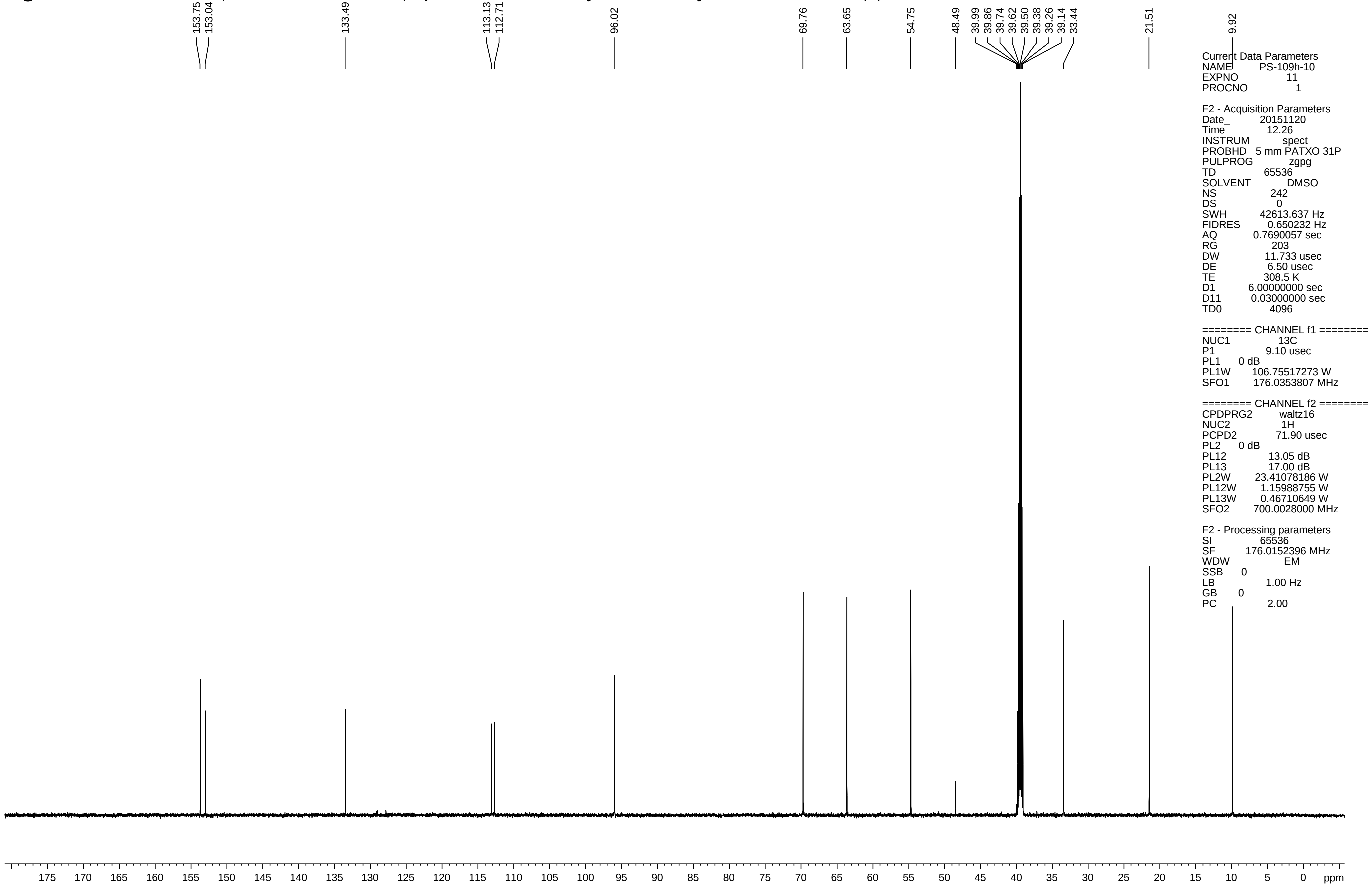

Figure S14. ^{1}H NMR (500 MHz, acetone-d_6) spectra of mactanamide (**6**)

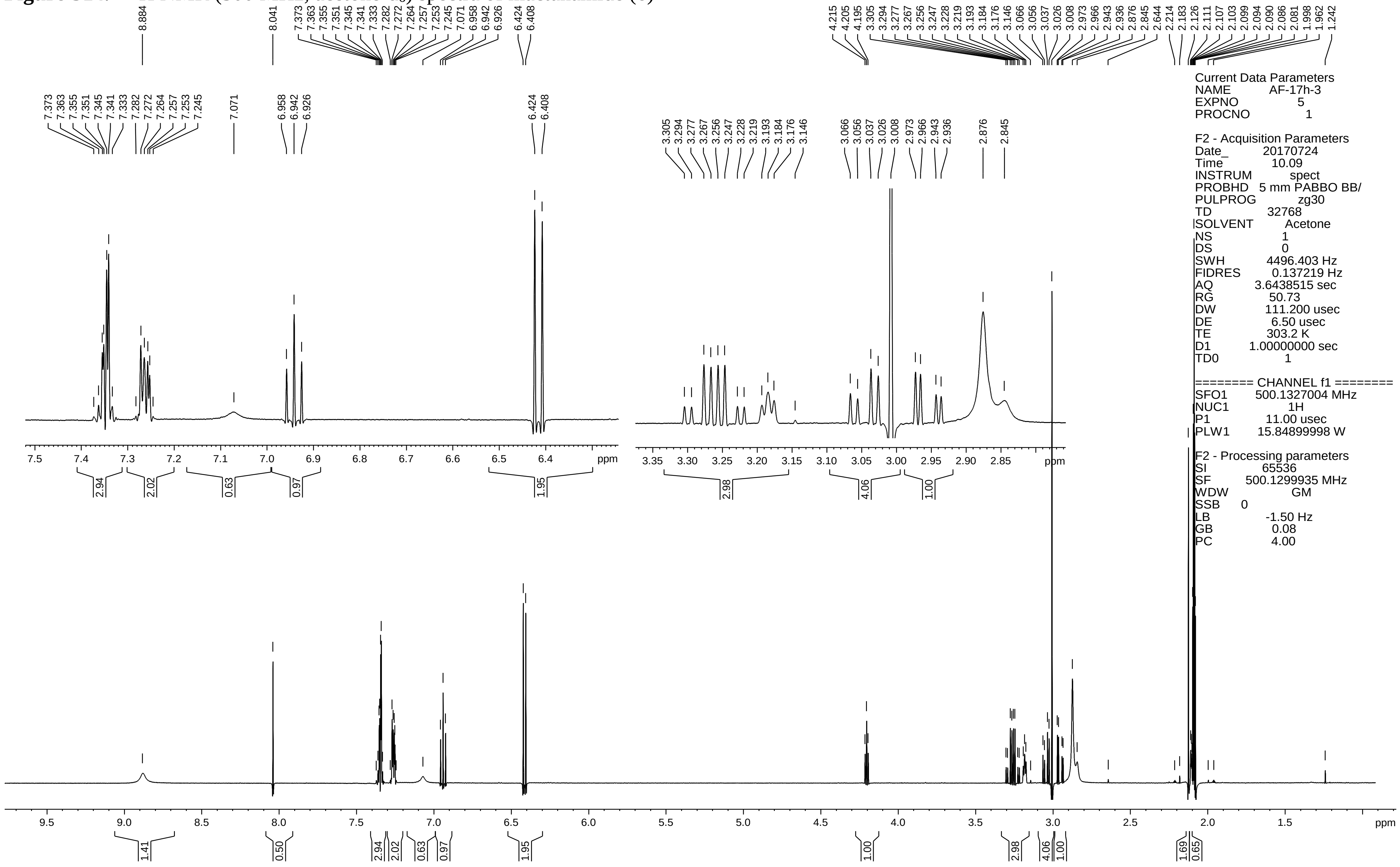

Figure S15. ^{13}C NMR (125 MHz, acetone-d_6) spectrum of mactanamide (**6**)

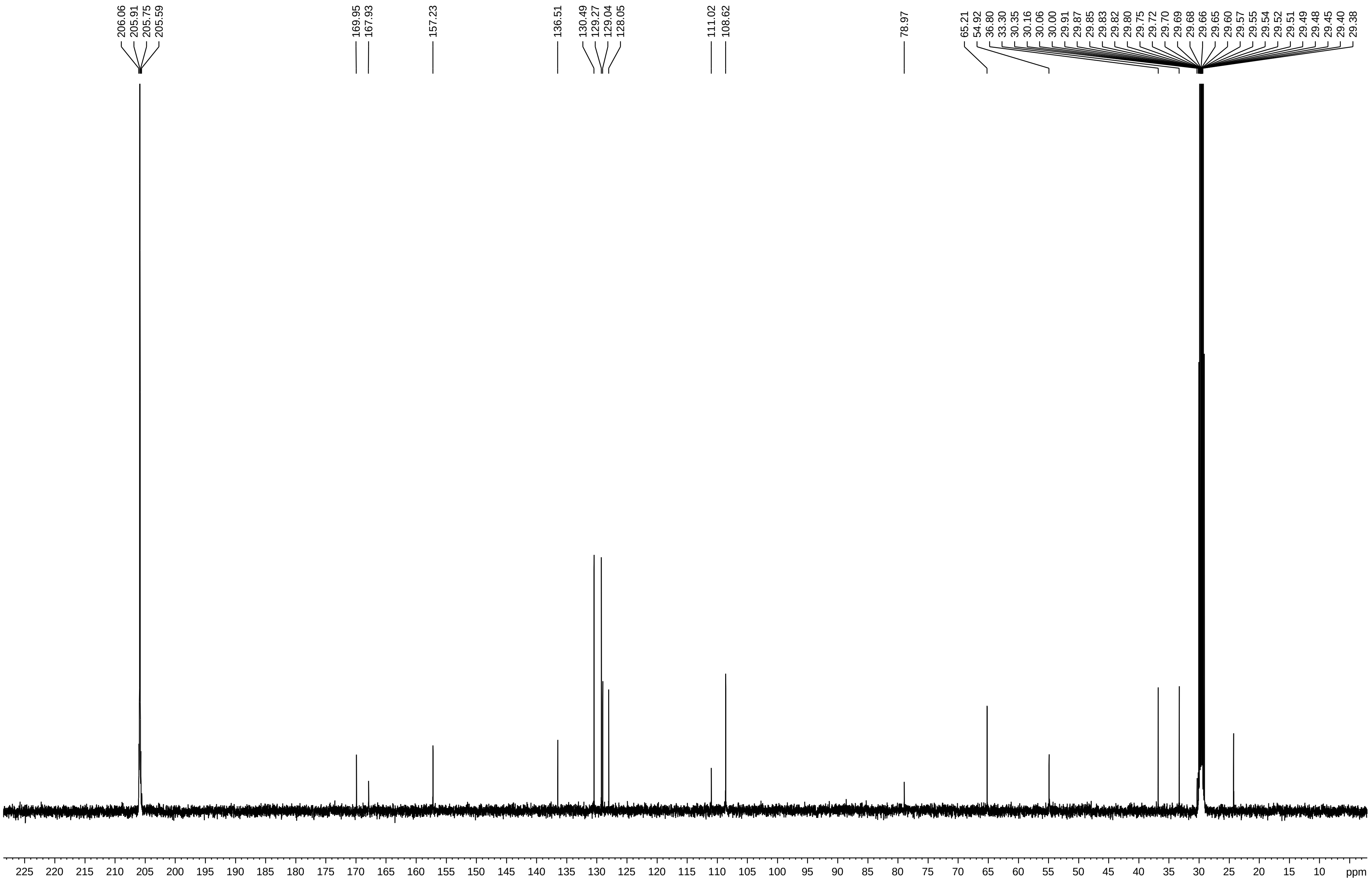

Figure S16. ROS formation in 6-OHDA- and PQ-treated Neuro2a cells

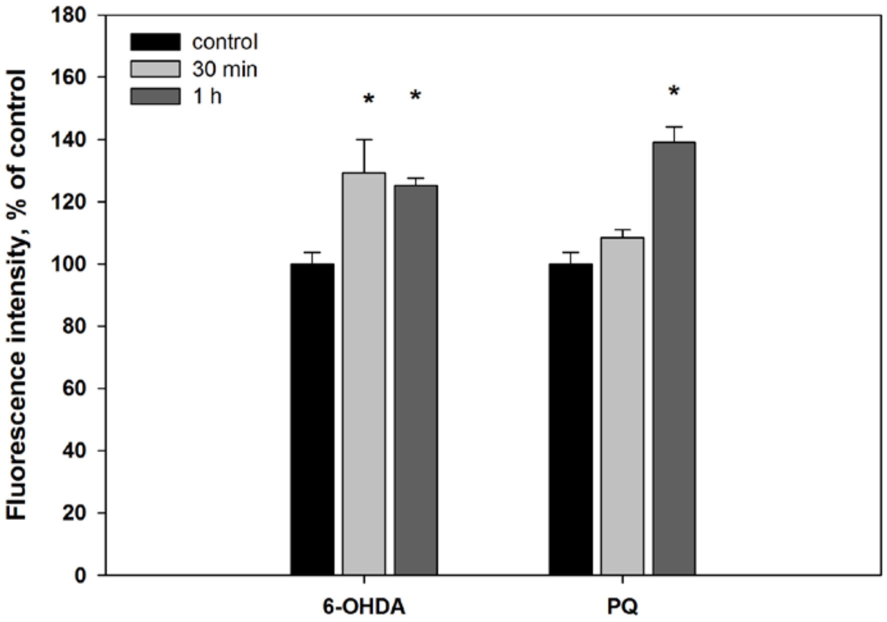

Supplement: Supplementary file 1 [file marinedrugs-16-00457-s001.pdf]
